# Progranulin Deficiency Induces Mitochondrial Dysfunction in Frontotemporal Lobar Degeneration with TDP-43 Inclusions

**DOI:** 10.3390/antiox12030581

**Published:** 2023-02-25

**Authors:** Guiomar Rodríguez-Periñán, Ana de la Encarnación, Fermín Moreno, Adolfo López de Munain, Ana Martínez, Ángeles Martín-Requero, Carolina Alquézar, Fernando Bartolomé

**Affiliations:** 1 Group of Neurodegenerative Diseases, Hospital Universitario 12 de Octubre Research Institute (imas12), 28041 Madrid, Spain; 2 Department of Molecular Biomedicine, Centro de Investigaciones Biológicas, Margarita Salas (CSIC), Ramiro de Maeztu 9, 28040 Madrid, Spain; 3 Neuroscience Area, Biodonostia Health Research Institute, Donostia-San Sebastián, 20014 Gipuzkoa, Spain; 4 Center for Biomedical Research in Neurodegenerative Diseases (CIBERNED), Biomedical Research Networking Centers (CIBER), Institute Carlos III, 28031 Madrid, Spain; 5 Neurology Department, Donostia University Hospital-OSAKIDETZA, 20014 Donostia-San Sebastián, Spain; 6 Neuroscience Department, University of the Basque Country (UPV/EHU), 20014 Donostia-San Sebastián, Spain; 7 Department of Medicine, Faculty of Medicine, Deusto University, 48007 Bilbao, Spain; 8 Department of Structural and Chemical Biology, Centro de Investigaciones Biológicas, Margarita Salas (CSIC), Ramiro de Maeztu 9, 28040 Madrid, Spain

**Keywords:** progranulin, FTLD-TDP, mitochondria, bioenergetics, mitophagy, autophagy

## Abstract

Loss-of-function (LOF) mutations in *GRN* gene, which encodes progranulin (PGRN), cause frontotemporal lobar degeneration with TDP-43 inclusions (FTLD-TDP). FTLD-TDP is one of the most common forms of early onset dementia, but its pathogenesis is not fully understood. Mitochondrial dysfunction has been associated with several neurodegenerative diseases such as Alzheimer’s disease (AD), Parkinson’s disease (PD) and amyotrophic lateral sclerosis (ALS). Here, we have investigated whether mitochondrial alterations could also contribute to the pathogenesis of PGRN deficiency-associated FTLD-TDP. Our results showed that PGRN deficiency induced mitochondrial depolarization, increased ROS production and lowered ATP levels in *GRN* KD SH-SY5Y neuroblastoma cells. Interestingly, lymphoblasts from FTLD-TDP patients carrying a LOF mutation in the *GRN* gene (c.709-1G > A) also demonstrated mitochondrial depolarization and lower ATP levels. Such mitochondrial damage increased mitochondrial fission to remove dysfunctional mitochondria by mitophagy. Interestingly, PGRN-deficient cells showed elevated mitochondrial mass together with autophagy dysfunction, implying that PGRN deficiency induced the accumulation of damaged mitochondria by blocking its degradation in the lysosomes. Importantly, the treatment with two brain-penetrant CK-1δ inhibitors (IGS-2.7 and IGS-3.27), known for preventing the phosphorylation and cytosolic accumulation of TDP-43, rescued mitochondrial function in PGRN-deficient cells. Taken together, these results suggest that mitochondrial function is impaired in FTLD-TDP associated with LOF *GRN* mutations and that the TDP-43 pathology linked to PGRN deficiency might be a key mechanism contributing to such mitochondrial dysfunction. Furthermore, our results point to the use of drugs targeting TDP-43 pathology as a promising therapeutic strategy for restoring mitochondrial function in FTLD-TDP and other TDP-43-related diseases.

## 1. Introduction

Heterozygous loss-of-function (LOF) mutations in granulin (*GRN*) gene leading to progranulin (PGRN) happloinsufficiency have been identified as a major cause of familial frontotemporal lobar degeneration with TDP-43 accumulation (FTLD-TDP) [1,2,3,4,5,6]. FTLD-TDP patients exhibit behavioral changes and language difficulties associated with the neuronal death in the frontal and temporal lobar brain cortex. Currently, the mechanisms by which reduced levels of PGRN lead to neurodegeneration are still unknown. Mitochondrial dysfunction has been shown to contribute to neuronal death in neurodegenerative disorders such as Alzheimer’s disease (AD), Parkinson’s disease (PD) and amyotrophic lateral sclerosis (ALS) [7,8,9,10,11]. Recent evidence has shown that PGRN plays an important role in regulating mitochondrial homeostasis and activity [12,13]. Furthermore, previous results from our lab showed that PGRN insufficiency regulated the intrinsic/mitochondrial apoptosis pathway in *GRN* knockdown (KD) SH-SY5Y neuroblastoma cells [14,15] and peripheral cells from FTLD-TDP patients carrying a LOF *GRN* mutation (c.709-1G > A) [16]. Altogether, this evidence suggests that mitochondrial dysfunction could also contribute to neurodegeneration in FTLD-TDP linked to PGRN deficiency. Therefore, understanding the molecular mechanisms by which PGRN deficiency leads to mitochondrial dysfunction in FLD-TDP may lead to the identification of new therapeutic strategies.

In this work, we have investigated the effect of PGRN deficit on mitochondrial function, dynamics and degradation, using a cellular model of FTLD-TDP with PGRN deficiency (SH-SY5Y *GRN* KD cells) and lymphoblasts from FTLD-TDP patients carrying a LOF mutation in the *GRN* gene (c.709-1G > A). Our results showed that PGRN deficiency induced mitochondrial damage in both FTLD-TDP models. Interestingly, we found that because of the autophagy impairment associated with PGRN loss, the damaged mitochondria in PGRN-deficient cells failed to be degraded, leading to mitochondrial accumulation. Importantly, the treatment with TDP-43 phosphorylation inhibitors rescued mitochondrial function in *GRN* KD cells, suggesting a key role for TDP-43 pathology in the mitochondrial dysfunction observed in PGRN-deficient cells. Together, our results indicate that mitochondrial impairment might contribute to neuronal death in FTLD-TDP associated with LOF *GRN* mutations and that modulating TDP-43 phosphorylation might represent a good therapeutic approach to rescue the mitochondrial dysfunction and the consequent neurodegeneration in FTLD-TDP and other TDP-43 proteinopathies.

## 2. Materials and Methods

### 2.1. Cell Lines Culture and Treatments

The control and stable *GRN* KD human neuroblastoma SH-SY5Y cells (Clone # 207) were a generous gift from Drs. Alvin P. Joselin and Jane Y. Wu from the Center for Genetic Medicine (Northwestern University, Chicago, IL, USA). These lines were generated using the pSUPERIOR RNAi construct containing a sequence of 19 nucleotides targeting human *GRN*, as was previously described [14,17]. Cells were cultured in Dulbecco’s Modified Eagle Medium (DMEM; Thermo Fisher Scientific, Waltham, MA, USA), supplemented with 10% (*v*/*v*) heat-inactivated fetal bovine serum (FBS; Thermo Fisher Scientific, MA, USA) and 1% penicillin/streptomycin (P/S; Thermo Fisher Scientific, MA, USA). When necessary, DMEM was supplemented with other compounds as follows: (i) to block autophagy, SH-SY5Y cells were cultured for 12 h in DMEM containing 100 nM of V-ATPase inhibitor bafilomycin A1 (BafA1; Sigma-Aldrich, MO, USA) and (ii) to block TDP-43 phosphorylation, SH-SY5Y cells were cultured for 48 h in DMEM containing the casein kinase 1 (CK1) inhibitors IGS-2.7 and IGS-3.27 (5 μM). These two small molecules were synthetized in our laboratories according to procedures previosly described [18,19].

The lymphoblastic cell lines used in this study (Table 1) were generated in our laboratory by infecting peripheral blood lymphocytes from FTLD-TDP patients and control subjects with Epstein Barr virus (EBV), as previously described [20]. Lymphoblastic cell lines were grown in suspension using RPMI-1640 medium supplemented with 1% P/S and 10 % (*v*/*v*) FBS. PGRN levels were measured in plasma samples from all subjects involved in this study using the PGRN ELISA kit AG-45A-0018YEK-KI01 (AdipoGene, Füllinsdorf, Switzerland), following the manufacturer’s protocol. Peripheral blood samples of all the individuals enrolled in this studio were taken after obtaining written informed consent of the patients or their relatives. All study protocols were approved by the Donostia Hospital and the Spanish Council of Higher Research Institutional Review Board (01/01/2006) and are in accordance with National and International Guidelines (Declaration of Helsinki).

### 2.2. Cell Lysates and Immunoblot

To extract the proteins, cells were harvested, washed with phosphate buffered saline (PBS; Thermo Fisher Scientific, MA, USA) and lysed with NP-40 lysis buffer (50 mM Tris pH 7.4, 150 mM NaCl, 50 mM NaF, 1% Nonidet P40) containing mini-complete protease and phosphatase inhibitor cocktails (Roche). Lysates were centrifuged at 15,000 rpm for 15 min at 4 °C to obtain the supernatant containing soluble proteins. To perform the immunoblot, protein concentration was determined by bicinchoninic acid (BCA) assay (Thermo Fisher Scientific, MA, USA) and then equivalent amounts of protein were separated on 4–12% SDS-PAGE gels (Thermo Fisher Scientific, MA, USA) and transferred onto PVDF membranes (Millipore, MA, USA). Membranes were blocked at room temperature with 5% non-fat milk or 5% BSA and incubated at 4 °C overnight with the following primary antibodies: rabbit anti-Progranulin (PGRN; 1:500, Abcam, Cambridge, UK), rabbit anti-TDP-43 (10782-2-AP, 1:2000 Proteintech, IL, USA), rabbit anti-phosphorylated TDP-43 (23309-1-AP, 1:500 Proteintech, IL, USA), rabbit anti-TDP-43 C-terminal (12892-1-AP, 1:1000 Proteintech, IL, USA), mouse anti-complex V-β subunit (CxVβ, 1:1000; Abcam, Cambridge, UK), rabbit anti-peroxisome proliferator-activated receptor γ co-activator 1α (PGC1α, 1:200; Santa Cruz Biotechnologies, CA, USA), mouse anti-Mitofusin1 (Mfn1, 1:1000; Abcam, Cambridge, UK), mouse anti-Mitofusin2 (Mfn2, 1:1000; Abcam, Cambridge, UK), mouse anti-mitochondrial Dynamin-like GTPase (Opa1, 1:1000; Novus Biologicals, CO, USA), rabbit anti-Dynamin-related protein 1 (Drp1, 1:1000, Cell Signalling Technology, MA, USA), rabbit anti-mitochondrial Fission protein 1 (FIS1, 1:1000, Abcam, Cambridge, UK), mouse anti-β-actin HRP (1:25,000; Abcam, Cambridge, UK), rabbit anti-p62/SQSTM1 (1:20,000. Abcam, Cambridge, UK) and rabbit anti-LC3 (1:1000, Novus Biologicals, CO, USA). Prior to band visualization, the membranes were incubated for 1 h at room temperature with species-specific horseradish peroxidase conjugated secondary antibodies as follows: goat anti-rabbit HRP secondary antibody (1:5000; Thermo Fisher Scientific, MA, USA) and goat anti-mouse HRP secondary antibody (1:5000; Abcam, Cambridge, UK). Bands were visualized by chemiluminescent substrate detection (ECL Clarity; Bio Rad, CA, USA) system using the ImageQuant LAS 4000 system (GE Healthcare, IL, USA). Images showing gels/blots and fluorescence images are in compliance with the digital image and integrity policies. Protein band densities were quantified using Fiji software (https://imagej.net/ (accessed on 11 January 2023)).

### 2.3. Quantitative Real-Time PCR

Total RNA from lymphoblasts was extracted using Trizol (Invitrogen, Alcobendas, Madrid, Spain) and was used to perform a qPCR as previously described [21]. Briefly, RNA was treated with DNase I Amplification Grade (Invitrogen, Alcobendas, Madrid, Spain) and then transcribed into cDNA using the Superscript III Reverse Transcriptase kit (Invitrogen, Alcobendas, Madrid, Spain). Quantitative real-time polymerase chain reaction (PCR) was performed in triplicates using the TaqMan Universal PCR MasterMix No Amperase UNG reagent (Applied Biosystems, Alcobendas, Madrid, Spain) and the Bio-Rad iQ5 system with a thermal profile of an initial 5 min melting step at 95 °C followed by 40 cycles at 95 °C for 10 s and 60 °C for 60 s. *GRN* relative messenger RNA (mRNA) levels were normalized to β-actin expression using the simplified comparative threshold cycle delta–delta CT method (2-[ΔCT PGRN -ΔCT actin]). Primers were designed using the Universal ProbeLibrary for Human (Roche Applied Science, Madrid, Spain) and used at a final concentration of 20 μM (*GRN* primers: 5′-tctgtagtctgagcgctaccc-3′ and 5′-agggtccacatggtctgc-3′; *β-actin* primers: 5′-ccaaccgcgagaagatga-3′ and 5′-ccagaggcgtacagggatag-3′).

### 2.4. Cell Viability and Apoptosis Measurement

Cell viability was assessed using the MTT assay. Cells were seeded in triplicate in 96-well plates, and 72 h later, 10 µL of 5 mg/mL 3-(4,5-dimethylthiazol-2-yl)-2,5 diphenyltetrazolium bromide reactive (MTT; Sigma-Aldrich, St. Louis, MO, USA) was added to each well containing 100 µL of media and incubated for 4 h at 37 °C to allow the viable cells to reduce the MTT to formazan. Purple formazan crystals were then dissolved in 100 µL DMSO and the absorbance was measured at 595 nm using a microplate reader (EnSpire, PerkinElmer Waltham, MA, USA).

Apoptosis was assessed by the microscopic examination of nuclei morphology. To do so, cells were stained with DAPI (4,6-diamidino-2 phenylindole) (Thermo Fisher Scientific, MA, USA) and then imaged using a Leica TCS SP5 confocal microscope (Leica Microsystems, Wetzlar, Germany) with an excitation peak at 359 nm and an emission peak at 457 nm. Cells displaying highly condensed nuclei, or *pyknotic nuclei*, were considered apoptotic cells.

### 2.5. Measurement of Mitochondrial Membrane Potential (ΔΨ_m_)

Mitochondrial membrane potential (ΔΨ_m_) was analyzed using the cell-permeant fluorescent dyes tetramethylrhodamine ethyl ester (TMRE, Thermo Fisher Scientific, MA, USA) and tetramethylrhodamine methyl ester (TMRM, Thermo Fisher Scientific, MA, USA), according to previously established protocols [22,23]. Briefly, cells were seeded either in 96-well plates or in 6-well plates on 25 mm coverslips and 48 h after seeding, cells were incubated with 40 nM TMRE or TMRM in a HEPES-buffered salt solution (HBSS) (156 mM NaCl, 3 mM KCl, 2 mM MgSO_4_, 1.25 mM KH_2_PO_4_, 2 mM CaCl_2_, 10 mM glucose and 10 mM HEPES; pH adjusted to 7.35 with NaOH) for 40 min at 37 °C. Then, TMRE/TMRM fluorescence was assessed using either a POLARstar Galaxy spectrofluorimeter (BMG Labtechnologies, Offenburg, Germany) or a Zeiss 510 confocal microscope equipped with META detection system (Zeiss, Oberkochen, Germany) with 40× oil immersion objective. In both cases, excitation wavelength was 560 nm and emission was detected above 580 nm. Microscope images were analyzed using the Volocity software (Quorum Technologies, Ontario, Canada) and TMRM values for untreated cells were set to 100%.

### 2.6. Reactive Oxygen Species (ROS) Measurement

Intracellular accumulation of ROS was determined using the fluorescent probe CM-H_2_DCFDA (Thermo Fisher Scientific, MA, USA). To do so, control and *GRN* KD SH-SY5Y cells were seeded in 96-well plates, and 48 h later, cells were loaded with 10 µM CM-H_2_DCFDA for 30 min. Fluorescence measurements were carried out using a POLARstar Galaxy spectrofluorimeter (BMG Labtechnologies, Offenburg, Germany). The Excitation wavelength used was 495 nm and emission was detected above 510 nm.

Mitochondrial ROS levels were measured using MitoSox (Thermo Fisher Scientific, MA, USA), a fluorogenic dye specifically targeted to mitochondria in living cells, which produces red fluorescence when oxidized by superoxide. SH-SY5Ycells were cultured in 6-well plates on 25 mm coverslips during 48 h and then incubated with 5 µM MitoSox in HBSS for 30 min at room temperature. Z-stack images were obtained using a Zeiss 510 confocal microscope equipped with META detection system (Zeiss, Oberkochen, Germany) and 40× oil immersion objective using an excitation wavelength of 510 nm. Emission was detected at 580 nm. Fluorescence intensity was quantified using the Volocity software (Quorum Technologies, Ontario, Canada).

### 2.7. Measurement of Cellular Oxygen Consumption

Cellular oxygen consumption rate (OCR) was measured using a Seahorse XF24 Extracellular Flux Analyzer (Seahorse, Agilent Technologies, CA, USA). A total of 20,000 control and *GRN* KD SH-SY5Y cells per well were plated in DMEM supplemented with 10% FBS and 1% P/S. Then, 24 h later, cell growing media was replaced by 25 mM glucose, 1 mM Pyruvate and 2 mM L-glutamine containing XF Base medium. Cells were incubated for 1 h in a CO_2_-free incubator at 37  °C allowing temperature and pH equilibration before loading into the XF24 analyzer. Mitochondrial function was determined through sequential addition of 1 µM oligomycin (Sigma-Aldrich, MO, USA), 0.5 µM carbonylcyanide-p-trifluoromethoxyphenylhydrazone (FCCP; Sigma-Aldrich, MO, USA) and 1 µM antimycin (Sigma-Aldrich, MO, USA)/1 µM rotenone (Sigma-Aldrich, MO, USA). This sequential addition allowed us to determine the basal oxygen consumption, oxygen consumption-linked to ATP synthesis (ATP), maximal respiration and the cellular spare capacity.

### 2.8. ATP Levels Measurement

In SH-SY5Y cells, ATP was measured using a FRET-based ATP-plasmid indicator (AT1.03 sensor) kindly provided by Dr. H. Imamura, following previously established protocols [24]. Briefly, control and *GRN* KD SH-SY5Y cells were seeded in 6-well plates on 25 mm coverslips and then transfected with the FRET-based ATP-plasmid indicator using Effectene transfection reagent (Qiagen, Hilden, Germany) according to the manufacturer’s instructions. One day after transfection, cell culture media were replaced with HBSS medium plus Ca^2+^ and Mg^2+^ and then cells were subjected to a time-dependent fluorescence imaging using a Zeiss 510 LSM confocal microscope with META detection system. Images were obtained using a 63× oil-immersion objective. Excitation of cyan fluorescent protein was 405 nm and emission was detected between 460 and 510 nm. Yellow fluorescent protein was excited using the 405 nm laser line and emission was detected using a band-pass filter from 515 to 580 nm. Minimal illumination intensity was kept to avoid phototoxicity (at 0.1–0.2% of laser output) and the pinhole was adjusted to give an optical slice of ~2 μm. The ratio-metric analysis of the yellow- and cyan-fluorescent proteins was assessed using the ZEN software (Zeiss, Oberkochen, Germany) and allowed the estimation of ATP kinetics within single cells.

In lymphoblasts, ATP basal levels were measured by using the Vialight plus assay kit (Lonza, Verviers, Belgium). This assay allows the measurement of ATP present in all metabolically active cells and is based upon the bioluminescent measurement of ATP. Lymphoblasts were seeded in 24-well plate and after 48 h, cells were lysed using the cell lysis buffer provided by the kit. Protein concentration of the cell lysates was estimated using the BCA assay (Thermo Fisher Scientific, MA, USA) and samples were diluted in cell lysis buffer to obtain 0.1 μg/μL protein concentration. Then, 100 μL of the protein solutions were transferred to each well of a 96-well plate containing 100 μL of ATP monitoring reagent plus (in triplicates). Plates were incubated for 2 min at room temperature and then luminescence was measured using an EnSpire microplate reader (PerkinElmer Waltham, MA, USA).

### 2.9. Mitochondrial Mass

Mitochondrial mass was measured using the cell permeable mitochondria-selective dye MitoTracker Green FM (Thermo Fisher Scientific, MA, USA). MitoTracker is a fluorescent dye that localizes to the mitochondrial matrix regardless of the mitochondrial membrane potential and covalently binds to mitochondrial proteins by reacting with free thiol groups of cysteine residues. *GRN* KD and control SH-SY5Y cells were seeded in 96- or 24-well plates. For this assay, the same number of *GRN* KD and control SH-SY5Y cells were seeded in each well. Then, 72 h later, SH-SY5Y cells were incubated for 15 min with 400 nM of MitoTracker Green FM and then fluorescence was determined by using either a confocal microscope Leica TCS SP5 (Leica Microsystems, Wetzlar, Germany) or a spectrofluorimeter (BMG Labtechnologies, Offenburg, Germany). In both cases, excitation wavelength was 490 nm and emission was detected at 516 nm.

Mitochondrial mass was also assessed by analyzing the levels of the mitochondrial structural protein complex V-β subunit (CxVβ) by immunoblotting or immunofluorescence, as previously described [25,26,27]. Briefly, to assess mitochondrial mass by immunoblotting, equal amounts of total protein were loaded for all the samples and CxVβ levels were normalized using the cytoskeleton protein β-actin. To assess mitochondrial mass by immunofluorescence, cells were seeded in 24-well plates on 13 mm coverslips and stained using an anti-CxVβ antibody. Then, Z-stack images were obtained using a Zeiss 510 LSM confocal microscope with META detection system with a 40× oil immersion objective, which were analyzed using the Volocity software (Quorum Technologies, Ontario, Canada), which allowed us to visualize, analyze and quantify 3D fluorescence images.

### 2.10. Immunofluorescence and Colocalization Analysis

SH-SY5Y cells were seeded at an initial density of 1 × 10^5^ cells/well in 24-well plates containing 12 mm coverslips, and 48 h after seeding, half of the coverslips were treated with BafA1 (100 nM) for 12 h and then fixed with 4% PFA. For the immunostaining, cells were blocked with 2% BSA in PBS, permeabilized with 0.1% saponin, incubated with primary antibodies (rabbit anti-p62/SQSTM1, 1:200 and mouse anti-CxVβ, 1:200) at room temperature. Then, 1 h later, cells were incubated with the corresponding secondary antibodies (Alexa-fluor 488 goat anti-rabbit, 1:200, and Alexa-fluor 568 goat anti-mouse, 1:200, both from Thermo Fisher Scientific, MA, USA) for 45 min at room temperature. Preparations were mounted using proLong Gold antifade mountant with DAPI (4′,6-diamidino-2-phenylindole) (Thermo Fisher Scientific, MA, USA) and visualized using a Zeiss 510 LSM confocal microscope with META detection system and a 40× oil immersion objective. Fluorescence intensity and colocalization were analyzed using Volocity software (Quorum Technologies, Ontario, Canada). Pearson’s correlation coefficient was used to estimate the colocalization between green and red channels.

### 2.11. Statistical Analysis

Student’s *t* test, one-way and two-way analysis of variance (ANOVA) statistical analyses were performed using GraphPad Prism 6. Bonferroni’s analysis was used to analyze the statistical significance between multiple groups. Plots show means ± Standard Error of the Mean (SEM) of all experiments performed. Differences were considered statistically significant when *p* < 0.05.

## 3. Results

### 3.1. GRN KD SH-SY5Y Cells Recapitulate Pathological Characteristics of FTLD-TDP

**Figure 1:**
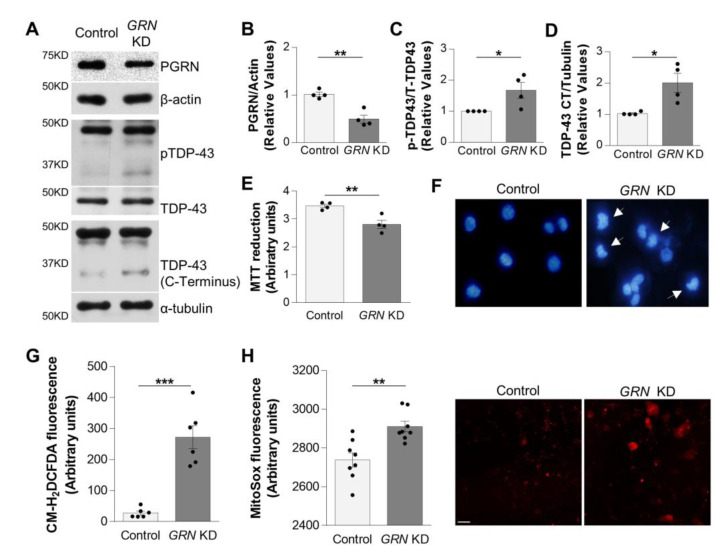
PGRN insufficiency induces TDP-43 pathology, apoptotic cell death and oxidative stress in SH-SY5Y cells. (**A**–**D**) Representative immunoblots and quantification showing that *GRN* KD SH-SY5Y cells exhibited increased TDP-43 phosphorylation at S409/410 residues and accumulation of C-Terminal fragments of TDP-43 protein. Plots represent the average ± SEM of the protein levels in four independent experiments. (**E**) Control and *GRN* KD SH-SY5Y cells were seeded in triplicate at same confluency (50,000 cells/well) on 96-well plates. Then, 72 h after seeding, cell viability was measured using MTT colorimetric assay. Plot represents the average ± SEM of four independent experiments. (**F**) Control and *GRN* KD SH-SY5Y cells were seeded at the same confluency on glass coverslips and 72 h later the apoptotic cell death was assessed by visualizing pyknotic nuclei (white arrows) using DAPI staining and fluorescence microscopy. (**G**) Control and *GRN* KD SH-SY5Y cells were seeded in triplicate at the same density in 96-well plates (50,000 cells/well) and 72 h later cytosolic ROS levels were assessed by incubating the cells with the oxidative stress indicator CM-H_2_DCFDA and measuring its fluorescence using a fluorescent microplate reader. Plot represents the average ± SEM of six independent experiments. (**H**) Control and *GRN* KD were seeded in coverslips and 72 h later mitochondrial ROS was assessed by staining the cells with MitoSOX Red reagent and measuring red fluorescence using a confocal microscope. Plot represents the average ± SEM eight independent measurements. Right panel shows representative images of MitoSOX staining in control and *GRN* KD SH-SY5Y cells. Scale bar = 22 µM. For all the experiments, the statistical analysis was performed using Student’s *t*-test. * *p* < 0.05; ** *p* < 0.01; *** *p* < 0.001.

Previous reports have demonstrated that PGRN depletion induces cytosolic TDP-43 accumulation in several cell models [18,28,29], suggesting that PGRN-deficient cells could be used to study FTLD-TDP. Here, we have investigated if *GRN* KD SH-SY5Y cells also recapitulate key aspects of FTLD-TDP pathophysiology such as TDP-43 phosphorylation/cleavage, neuronal death and oxidative stress [30,31,32]. We found that *GRN* depletion in SH-SY5Y cells led to increased TDP-43 protein phosphorylation at S409/410 and cleavage into 25 kDa C-terminal fragments (Figure 1A–D and Figure S1). Furthermore, *GRN* KD cells exhibited decreased cell viability (Figure 1E) and increased apoptosis (Figure 1F), as was indicated by the presence of morphological features of apoptotic cell death such as increased chromatin condensation and the formation of pyknotic nuclei (Figure 1F). To determine if *GRN* deficiency induced changes in oxidative stress status in SH-SY5Y cells, we assessed the cytosolic and mitochondrial levels of reactive oxygen species (ROS) by measuring fluoresce of CM-H_2_DCFDA and MitoSox probes, respectively. Both cytosolic (Figure 1G) and mitochondrial (Figure 1H) ROS production were increased in PGRN-deficient cells, compared with control SH-SY5Y cells. Together, these results indicate that *GRN* KD SH-SY5Y cells are an adequate model to study FTLD-TDP, as they mimic some of the main hallmarks of FLTD-TDP.

### 3.2. PGRN Insufficiency Impairs Mitochondrial Bioenergetics in SH-SY5Y Cells and FTLD-TDP Patient’s Lymphoblasts

**Figure 2:**
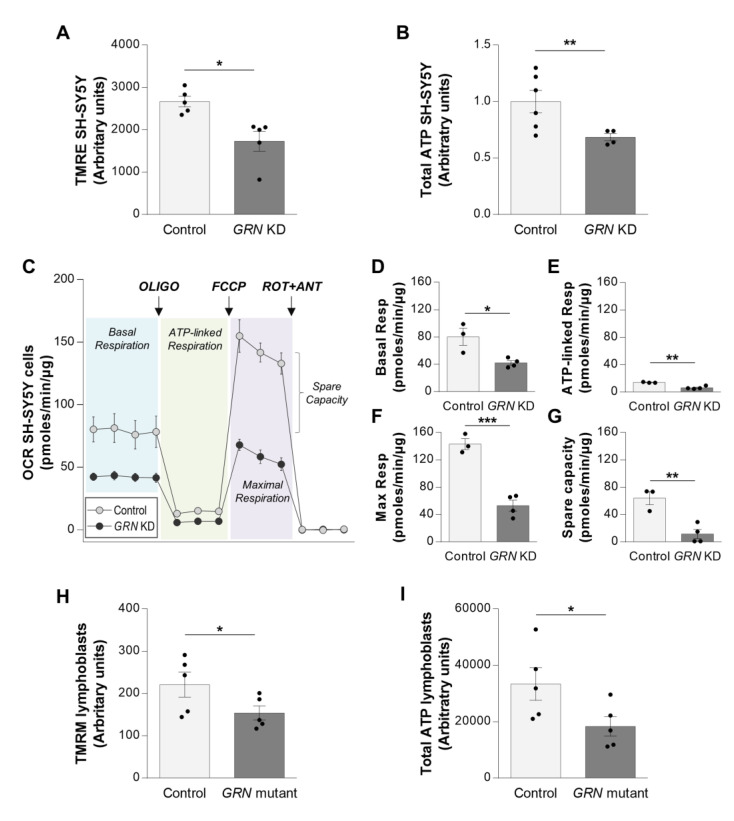
PGRN insufficiency reduces mitochondrial membrane potential, decreases ATP production and impairs mitochondrial respiration in SH-SY5Y cells and FTLD-TDP patients’ derived lymphoblasts. (**A**) Control and *GRN* KD SH-SY5Y cells were seeded in triplicate at the same confluency (50,000 cells/well) on 96-well plates and 48 h later cells were labeled with TMRE for 30 min to assess the mitochondrial membrane potential. TMRE fluorescence was measured using a plate reader (Ex/Em = 549/575 nm). Plot represents the average ± SEM of five independent experiments. (**B**) Total ATP measurements in control and GRN KD SH-SY5Y cells using a fluorescent FRET-based ATP-plasmid indicator. Fluorescent measurements were performed using a confocal microscope and a 63× oil-immersion objective (Ex/Em cyan = 405/460–510 nm; Ex/Em yellow = 405/515–580 nm). Plot represents average ± SEM of ATP measurements in control and *GRN* KD cells. (**C**) Plot summarizing the averaged traces of oxygen consumption rate (OCR) measurements in control and *GRN* KD SH-SY5Y cells, performed using Seahorse XF24 Extracellular Flux Analyzer (Oligo: Oligomycin; FCCP: Carbonyl cyanide 4-(trifluoromethoxy)phenylhydrazone; ROT: Rotenone; ANT: Antimycin). (**D**–**G**) Individual plots showing the average ± SEM of basal respiration (basal resp), ATP-linked respiration (ATP-linked Resp), maximal respiration capacity (Max Resp Capacity) and Spare capacity. (**H**) Lymphoblasts derived from five healthy individuals and five FTLD-TDP patients carrying a LOF mutation in the *GRN* gene were seeded in triplicate at 1 × 10^6^ cells/mL and 48 h later the mitochondrial membrane potential was measured by measuring TMRM fluorescence using a plate reader (Ex/Em = 549/575 nm). Plot represents the average ± SEM of TMRM fluorescence in all individuals. (**I**) Plot summarizing the measurement of ATP levels in lymphoblastoid cell lines generated from five healthy individuals and five FTLD-TDP patients carrying the same LOF *GRN* mutation. Lymphoblasts were seeded in triplicate at 1 × 10^6^ cells/mL and 48 h later ATP bioluminescence was assessed. Plot represents the average ± SEM of ATP bioluminiscence in all individuals. For all the experiments, the statistical analysis was performed using Student’s *t*-test. * *p* < 0.05; ** *p* < 0.01; *** *p* < 0.001.

Because mitochondria play a key role in ROS production and apoptotic cell death, we studied the mitochondrial function in our in vitro *GRN* KD cellular model. To do so, we analyzed the mitochondrial membrane potential (ΔΨ_m_), which reflects the mitochondrial health and function, in control and *GRN* KD SH-SY5Y cells using the TMRE probe. TMRE is a cell permeant, positively charged fluorescent dye that accumulates in active mitochondria. *GRN* KD cells exhibited a significant reduction of the TMRE signal (Figure 2A), indicating that PGRN deficiency induced mitochondrial depolarization. Consistent with the reduced ΔΨ_m_, we found that *GRN* KD cells exhibited reduced mitochondrial ATP levels (Figure 2B).

To further investigate how PGRN deficiency affects mitochondrial bioenergetics, we estimated the oxygen consumption rate (OCR) using the Seahorse XF analyzer (Figure 2C). *GRN* KD cells showed reduced basal OCR (Figure 2D). Consistent with the above results, after F_o_F_1_-ATP synthase inhibition with oligomycin (Figure 2C) PGRN-deficient cells demonstrated lower ATP production linked to respiration (Figure 2E). In addition, *GRN* KD cells displayed lower maximal respiration (Figure 2F) and spare capacity (Figure 2G), both obtained after addition of the mitochondrial uncoupler FCCP (Figure 2C). Together, these findings indicate that PGRN deficiency could be associated with reduced activity or lack of substrates for the mitochondrial respiratory complexes I or II.

We then investigated if the mitochondrial bioenergetics deficits observed in the *GRN* KD model could be extensible to FTLD-TDP patients. To do so, we used lymphoblastoid cell lines generated from FTLD-TDP patients carrying the c.709-1G > A heterozygous mutation in the *GRN* gene. This mutation is predicted to cause exon eight skipping, frameshift and premature translation termination, resulting in nonsense-mediated mRNA decay [33]. As expected, FTLD-TDP patients carrying this mutation exhibited decreased PGRN levels in plasma, compared with control subjects (Figure S2A). Furthermore, lymphoblastoid cell lines generated from the c.709-1G > A *GRN* mutation carriers exhibited reduced *GRN* mRNA and PGRN protein levels (Figure S2B–D). Importantly, similarly to *GRN* KD SH-SY5Y cells, lymphoblasts from FTLD-TDP patients carrying the c.709-1G > A *GRN* mutation exhibited depolarized mitochondria (Figure 2H) and reduced ATP levels when compared with lymphoblasts from healthy subjects (Figure 2I). These observations suggest that mitochondrial impairment might be a pathological feature of FTLD-TDP.

### 3.3. Progranulin Deficiency Increases Mitochondrial Mass

**Figure 3:**
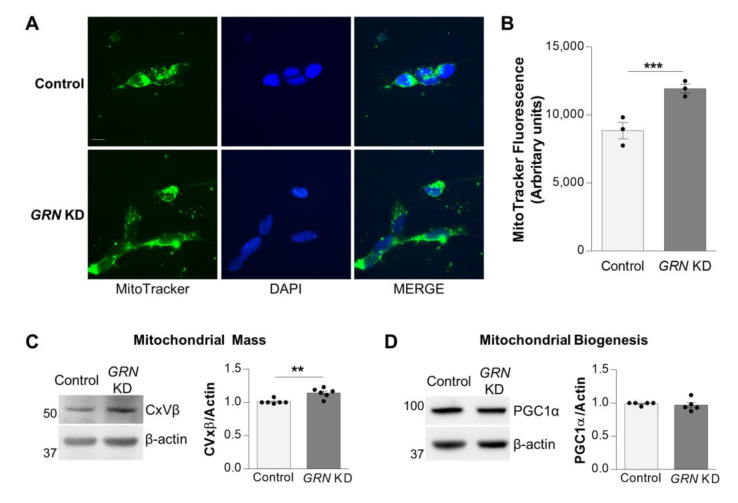
PGRN deficiency increases mitochondrial mass in SH-SY5Y cells. (**A**) Representative image of MitoTracker Green fluorescence in control and *GRN* KD SH-SY5Y cells. Fluorescence measurement was performed using a fluorescent confocal microscope and 63× oil-immersion objective. Scale bar = 9 µm. (**B**) Quantification of MitoTracker Green fluorescence in control and *GRN* KD SH-SY5Y cells. Fluorescence was assessed using a fluorescent microplate reader (Ex/Em = 490/516 nm). Plot represents the average ± SEM of three independent experiments. (**C**) Representative immunoblot showing the levels of the β subunit of mitochondrial Complex V (CxVβ) as a measurement of mitochondrial mass. β-actin was used as a loading control. Plot represents the average ± SEM of six independent measurements of CxVβ levels. (**D**) Representative immunoblot showing the levels of the mitochondrial biogenesis marker PGC1α. Plot represents the average ± SEM of five independent measurements of PGC1α levels. β-actin was used as a loading control. For all the experiments, the statistical analysis was performed using Student’s *t*-test. ** *p* < 0.01; *** *p* < 0.001.

To explore whether the impairment of mitochondrial bioenergetics in *GRN* KD cells could be explained by a reduced amount of mitochondria, we measured mitochondrial mass in control and PGRN-deficient cells. Interestingly, *GRN* KD SH-SY5Y cells exhibited higher mitochondrial mass as assessed using the MitoTracker Green FM fluorescence signal (Figure 3A,B). The increased mitochondrial mass in PGRN-deficient cells was then validated by immunoblot by measuring the levels of the mitochondrial structural protein complex V-β subunit (CxVβ) (Figure 3C and Figure S3A) and normalized by the levels of the cytoskeleton protein β-actin. Remarkably, the accumulation of mitochondria in *GRN* KD cells was not the result of increased mitochondrial biogenesis, as indicated by the presence of equal levels of the mitochondrial biogenesis marker PGC1α in both control and *GRN* KD cells (Figure 3D and Figure S3B).

These results suggested that the PGRN deficiency-induced increase in mitochondrial mass is not due to enhanced mitochondrial biogenesis but may be the result of impaired degradation of damaged mitochondria.

### 3.4. Impaired Autophagy in GRN KD Cells Blocks the Removal of Damaged Mitochondria

**Figure 4:**
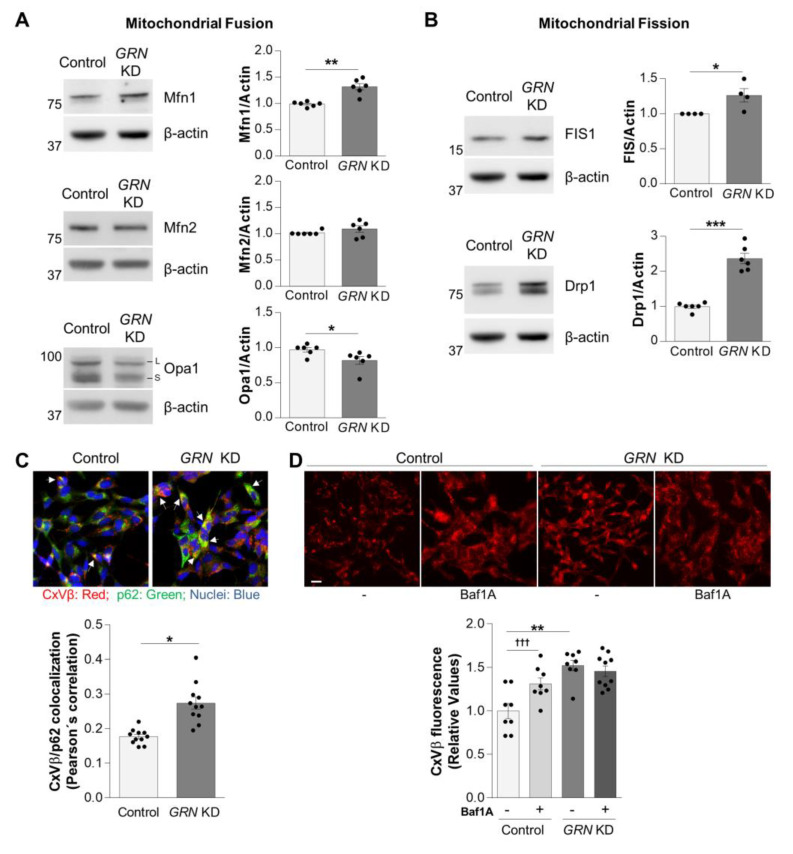
PGRN deficiency induces mitochondrial accumulation by blocking the lysosomal degradation of damaged mitochondria. (**A**) Representative immunoblots showing the levels of the mitochondrial fusion proteins mitofusin 1 (Mfn1), mitofusin 2 (Mfn2) and Opa1. β-actin was used as a loading control. Plots represent the average ± SEM of six independent measurements for each protein. (**B**) Representative immunoblots showing the levels of the mitochondrial fission proteins FIS1 and Drp1. β-actin was used as a loading control. Plots represent the average ± SEM of six independent measurements for each protein. The statistical analysis of protein level measurements was performed using Student’s *t*-test. * *p* < 0.05, ** *p* < 0.01; *** *p* < 0.001. (**C**) Microscopy images showing the colocalization of the mitochondrial marker CxVβ (in red) and the autophagic cargo adapter p62 (in green) in control and *GRN* KD SH-SY5Y cells. The colocalization between the two fluorophores was assessed by measuring the Pearson’s correlation coefficient. Plot represents the average ± SEM of the Pearson’s correlation coefficient measured in eleven images from three independent experiments. Statistical analysis was performed using Student’s *t*-test. * *p* < 0.05. (**D**) Fluorescence images showing the levels of CxVβ (in red) before and after the treatment with 100 nM bafilomycin A1 (BafA1) in control and *GRN* KD SH-SY5Y cells. CxVβ staining was used as a marker of mitochondrial accumulation. Scale bar = 9 µm. CxVβ fluorescence intensity was measured using Volocity software. Plot represents the average ± SEM of CxVβ fluorescence intensity measured in eight different images from three independent experiments. Statistical analysis was performed using two-way ANOVA’s tests followed by Bonferroni´s correction. ** *p* < 0.01 significant difference between untreated control and *GRN* KD cells. ^†††^ *p* < 0.001 significant difference between untreated control cells and BafA1 treated control cells.

Mitochondria are dynamic organelles that constantly fuse and divide. The processes of mitochondrial fusion and fission, known as mitochondrial dynamics, are key mechanisms for the mitochondrial quality control as they regulate the removal of damaged mitochondria by mitophagy. We studied the mitochondrial dynamics in the control and *GRN* KD SH-SY5Y cells by analyzing the levels of fusion and fission proteins such as mitofusin 1 and 2 (Mfn1-2), Opa1, FIS1 and Drp1 (Figure 4A,B, Figures S4 and S5). PGRN-deficient cells showed decreased levels of the mitochondrial fusion protein Opa1 (Figure 4A and Figure S4C) and increased levels of the mitochondrial fission proteins FIS1 and Drp1 in *GRN* KD cells, compared with control cells (Figure 4B and Figure S5), demonstrating an imbalance in the mitochondrial fusion/fission dynamics towards increased mitochondrial fission. To study whether the increased mitochondrial fission targeted the mitochondria for their disposal by mitophagy, we assessed the colocalization of mitochondria with the mitophagy marker p62 [34]. PGRN deficiency increased the colocalization of the mitochondrial marker CxVβ with p62 (Figure 4C and Figure S6), showing that damaged mitochondria were targeted for mitophagy in PGRN-deficient cells.

It has been previously demonstrated that PGRN plays an important role in regulating autophagy [35] and that PGRN depletion leads to autophagy blockage [36]. To address whether autophagy was also impaired in *GRN* KD SH-SY5Y cells, we measured autophagic flux by monitoring changes in the levels and localization of the autophagy adaptor p62 and the autophagosome marker LC3II (microtubule-associated protein 1 light chain 3B), before and after bafilomycin A1 (BafA1) treatment (Figure S7) [37]. Under basal conditions, *GRN* KD cells showed increased p62 levels together with decreased LC3II levels, compared with control cells (Figure S7A,B). Notably, when we added Baf1A, a V-ATPase inhibitor that inhibits autophagosome-lysosome fusion and blocks autophagosome degradation, the rate of LC3 II formation was lower in *GRN* KD cells than in control cells (Figure S7A). Together, these results suggest that *GRN* KD SH-SY5Y cells have reduced autophagy flux, probably associated with a failure in autophagosome formation. Thus, we asked if the mitochondrial accumulation associated with PGRN deficiency could be a consequence of the autophagy failure observed in *GRN*-deficient cells. To do so, we measured mitochondrial mass before and after blocking autophagy with BafA1. Consistent with the above results (Figure 3A–C), *GRN* KD cells showed increased CxVβ staining compared with control cells (Figure 4D). BafA1 treatment induced mitochondrial accumulation in control cells but did not modify CxVβ levels in *GRN* KD cells (Figure 4D). These results confirmed that mitochondrial accumulation in PGRN-deficient cells was a consequence of a general failure of autophagy.

### 3.5. Inhibition of TDP-43 Phosphorylation Restores Mitochondrial Bioenergetics in GRN KD Cells

**Figure 5:**
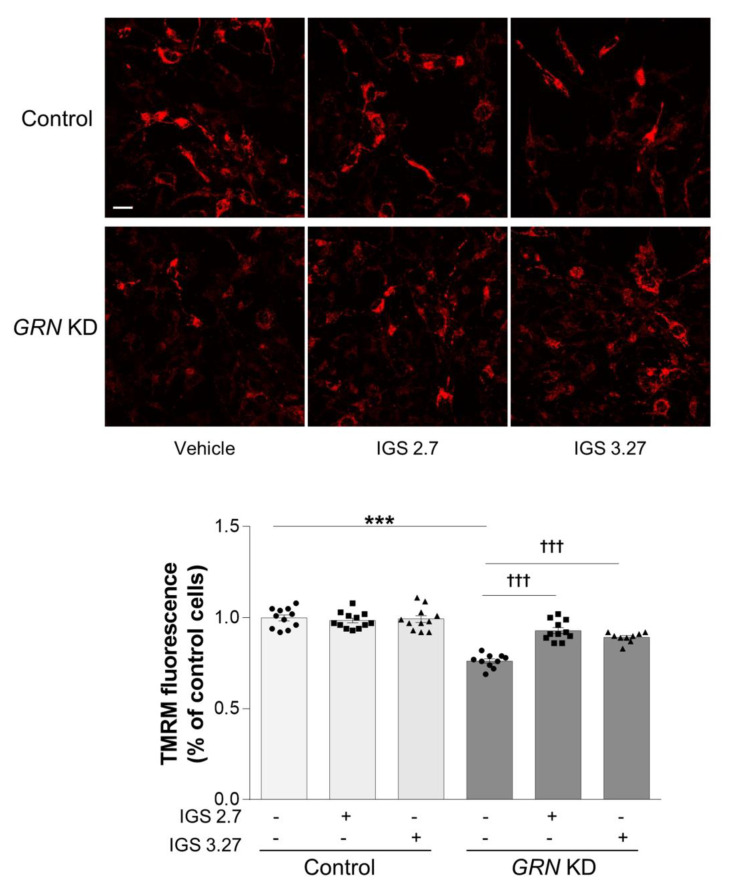
Treatment with pTDP-43 inhibitors rescues mitochondrial impairment in *GRN* KD SH-SY5Y cells. Control and *GRN* KD SH-SY5Y cells were seeded at the same confluency in coverslips and treated with 5 μM of two pTDP-43 inhibitors (IGS2.7 and IGS3.27) for 48 h. Mitochondrial membrane potential was measured by assessing TMRM fluorescence using a confocal microscope with 40× water-immersion objective (Ex/Em = 549/575 nm). Scale bar = 9 µm. Top panel shows representative images of TMRM staining in control and *GRN* KD cells before and after the treatment with both pTDP-43 inhibitors. Plot represents the average ± SEM of at least eleven images for each condition. The experiment was performed three times. Statistical analysis was performed using two-way ANOVA´s tests followed by Bonferroni´s correction. *** *p* < 0.001 significant difference between untreated control and *GRN* KD cells. ^†††^ *p* < 0.001 significant difference between untreated and treated *GRN* KD cells.

*GRN* KD SH-SY5Y cells accumulated S409/S410 phosphorylated C-terminal fragments of TDP-43 protein. It has been reported that casein kinase-1 δ (CK-1 δ) is the kinase that phosphorylates TDP-43 at these residues [38]. We previously developed two brain-penetrant CK-1δ inhibitors inhibitors (IGS2.7 and IGS3.27) and demonstrated that both compounds decreased TDP-43 phosphorylation and accumulation as well as prevented neuronal death in FTLD-TDP patient-derived lymphoblasts [18]. Because TDP-43 pathology in FTLD could be related to mitochondrial impairment [10,39,40,41], here we investigated if the inhibition of TDP-43 phosphorylation could have an effect in the mitochondrial bioenergetics of PGRN-deficient cells. Treatment with both CK-1δ inhibitors, IGS2.7 and IGS3.27, restored the ΔΨ_m_ in SH-SY5Y *GRN* KD cells, with no effect on control cells (Figure 5). These results suggested that the accumulation of phosphorylated forms of TDP-43 might be responsible for the mitochondrial impairment observed in *GRN* KD cells.

## 4. Discussion

Subjects carrying heterozygous *GRN* LOF mutations develop early onset FTLD-TDP, a neurodegenerative disease considered the second most common cause of dementia after AD [42]. However, the pathological mechanisms resulting in the clinical and cellular features of FTLD-TDP associated with *GRN* mutations are still not well understood. There is growing evidence that mitochondrial abnormalities are involved in the pathogenesis of common neurodegenerative diseases such as AD, PD and ALS [40,43,44,45], but little is known about the role of mitochondrial dysfunction in the pathogenesis of FTLD. This work was undertaken to investigate the link between mitochondrial dysfunction and FTLD-TDP associated with PGRN deficiency using a neuronal model of FTLD-TDP based on *GRN* gene silencing and lymphoblasts from FTLD-TDP patients carrying a *GRN* LOF mutation. Our results indicated that PGRN deficiency impaired mitochondrial bioenergetics in both the FTLD-TDP neuronal model and the FTLD-TDP patient’s derived lymphoblasts. Interestingly, previous reports from our lab demonstrated that PGRN deficiency induced alterations in mitochondrial/intrinsic apoptotic cell death [14,15,16]. Since apoptosis has been largely related to neuronal cell death in FTLD [46] it is likely that the mitochondrial impairment caused by PGRN deficiency may be one of the factors contributing to neuronal death in FTLD-TDP.

Further analysis of the bioenergetics status of our cell model showed that PGRN deficiency was associated with lower oxygen consumption. We also observed that *GRN* KD cells reached poor maximal respiration rates upon addition of the FCCP uncoupler, compared with control cells. These results along with the mitochondrial depolarization and the increased ROS production in *GRN* KD cells suggested that mitochondrial respiration might be inhibited in a complex I-dependent manner [47,48]. Previous reports demonstrated that in ALS and FTLD models, TDP-43 bound to the mitochondrial mRNA and impaired the expression of the complex I subunits ND3 and ND6 causing complex I disassembly. Similar to our results, in these reports the dysfunctional complex I resulted in increased ROS production, mitochondrial depolarization and reduced ATP production [40,41]. Together, this evidence demonstrates that in FTLD-TDP associated with PGRN deficiency, the inhibition of mitochondrial respiration may be due to a complex I deficiency caused by the accumulation of aberrant forms of TDP-43 protein. Interestingly, lymphoblasts from FTLD-TDP patients carrying a *GRN* LOF mutation also exhibited mitochondrial depolarization and reduced ATP levels, suggesting that the mitochondrial impairment could be a main feature of FTLD-TDP associated with *GRN* mutations.

Because mitochondria are crucial organelles for maintaining the physiological activity of cells, damaged mitochondria are rapidly degraded. Interestingly, PGRN-deficient cells showed accumulation of depolarized mitochondria. Among other causes, the accumulation of dysfunctional mitochondria could be associated with a defect in mitochondrial degradation. Mitochondrial degradation is regulated by mitochondrial dynamics and mitophagy [49]. Mitochondria are dynamic organelles that constantly undergo fission and fusion events. Whereas fusion helps maintain mitochondrial function, the fission process enables damaged mitochondria to be removed from the mitochondrial network for degradation by mitophagy. Our results showed that PGRN deficiency favored mitochondrial fission and the initiation of the mitophagy process, allocating the damaged mitochondria of *GRN* KD cells to degradation. These results agree with previous reports showing that depolarized mitochondria were degraded by mitophagy in vivo and in vitro [50,51,52]. However, although in PGRN-deficient cells the depolarized mitochondria initiated the mitophagy process, their degradation in the lysosomes was not completed. It has been demonstrated that PGRN regulates the autophagy–lysosomal pathway and that PGRN deficiency induces autophagy impairment [35,36]. In agreement with these reports, we found that PGRN deficiency induced autophagy failure in SH-SY5Y cells, implying that the accumulation of damaged mitochondria in *GRN* KD cells was due to a defect in the autophagy–lysosomal pathway and not to a failure of mitochondrial dynamics or mitophagy initiation.

Our findings of altered mitochondrial bioenergetics, dynamics and mitophagy in PGRN-deficient cells agree with previous reports showing that PGRN acts as a regulator of mitochondrial homeostasis [13] and activity [12]. However, these reports did not demonstrate whether the effect of PGRN in regulating mitochondrial function was direct or indirect. The fact that the inhibition of TDP-43 phosphorylation restored the mitochondrial membrane potential in *GRN* KD cells suggests that the accumulation of phospho-TDP-43 protein might be the responsible for the mitochondrial impairment observed in PGRN-deficient cells. Several studies using murine and cell models of ALS and FTLD overexpressing wild type or mutant TDP-43 have demonstrated a link between TDP-43 and mitochondria [10,39,40,41,53], describing that TDP-43 localizes to mitochondria, causing mitochondrial damage and a reduction in mitochondrial ATP synthesis [40,41]. Our results are consistent with these previously published data and support the hypothesis that TDP-43 pathology could play an important role inducing mitochondrial dysfunction in FTLD-TDP patients carrying LOF *GRN* mutations. On the other hand, PGRN deficiency might also affect the mitochondrial homeostasis through other pathways unrelated to TDP-43. For example, we previously reported an overactivation of Wnt signaling in *GRN* KD SH-SY5Y cells and lymphoblasts from FTLD-TDP patients carrying a LOF *GRN* mutation [15,54,55]. Both canonical and non-canonical Wnt signaling pathways have been implicated in mitochondrial dynamics and biogenesis [56,57,58]. Thus, the impairment of Wnt signaling in *GRN* KD cells might also contribute to the mitochondrial dysfunction. Interestingly, more recent reports have demonstrated a mitochondrial-initiated regulation of Wnt signaling [59,60], implying a bidirectional crosstalk between mitochondria and the Wnt pathway and suggesting that mitochondrial impairment might also be responsible for the alterations in Wnt pathway observed in PGRN-deficient cells.

**Figure 6:**
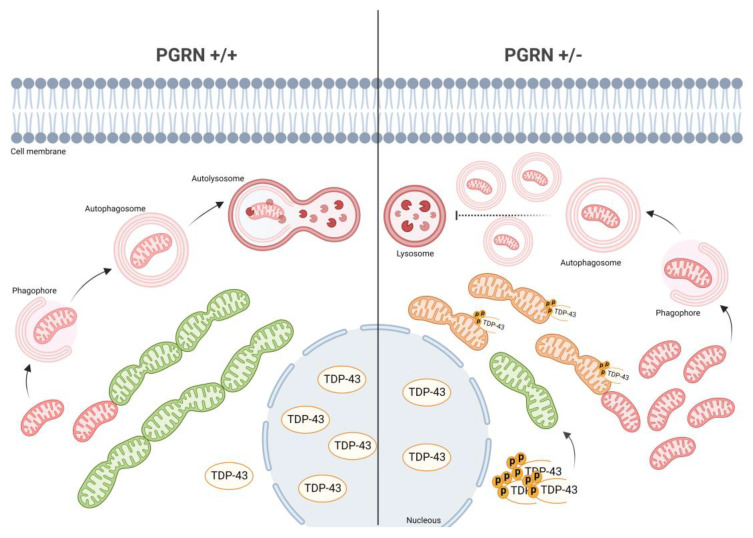
Diagram summarizing the role of PGRN insufficiency in regulating mitochondrial dysfunction and accumulation. In control cells with normal PGRN levels (PGRN +/+; left panel), TDP-43 protein has a predominant nuclear localization. In this case, the majority of mitochondria are healthy (in green) and impaired mitochondria (red) are released from the mitochondrial network by mitochondrial fission and then degraded in the lysosomes by mitophagy. On the other hand, PGRN-deficient neurons(PGRN +/−) display cytosolic accumulation of C-terminal fragments of phosphorylated TDP-43 protein. The accumulation of pTDP-43 seems to induce mitochondrial depolarization (orange mitochondria) and eventually mitochondrial damage. Damaged mitochondria (in red) are then excised from the mitochondrial network and engulfed by autophagosomes. PGRN-deficient cells show an impairment of the autophagy/lysosomes pathway and thus damaged mitochondria cannot complete the mitophagy process and accumulate in the cytosol. Arrows represent the autophagic flux direction. Dashed line indicate that in PGRN+/− cells the autofagosomes fail to be degraded in the lysosomes.

As is summarized in Figure 6, this study describes that the partial loss of PGRN provokes the imbalance of mitochondrial bioenergetics in a neuronal-like cell model and patient-derived lymphoblasts, which might contribute to the neuronal death in FTLD-TDP. Furthermore, it demonstrates that the autophagy failure associated with PGRN deficiency blocks the degradation of impaired mitochondria in the lysosomes leading to the aberrant accumulation of damaged mitochondria in FTLD-TDP cellular models. Our results also point out that the TDP-43 pathology contributes to the mitochondrial damage observed in *GRN* KD cells. Interestingly, the treatment with brain penetrant phospho-TDP-43 inhibitors restores mitochondrial function in PGRN-deficient cells, suggesting that the regulation of TDP-43 pathology might prevent neuronal death in FTLD-TDP and other TDP-43-related pathologies by reverting or preventing mitochondrial dysfunction.

## 5. Conclusions

This study demonstrates that PGRN deficiency causes mitochondrial dysfunction in an FTLD-TDP cell model and in lymphoblasts derived from FTLD-TDP patients, suggesting that mitochondria may be damaged in FTLD-TDP associated with LOF *GRN* mutations. Furthermore, we found that the autophagy failure associated with PGRN deficiency affects mitochondrial degradation, leading to the accumulation of damaged mitochondria. Importantly, our results show that the treatment with phospho-TDP-43 inhibitors restores mitochondrial function in PGRN-deficient cells, suggesting that the mitochondrial depolarization could be a consequence of TDP-43 pathology. Furthermore, this study points to the use of drugs targeting TDP-43 as promising therapies to restore mitochondrial function in FTLD-TDP and other TDP-43-related diseases.

## Figures and Tables

**Table 1:** Demographic characteristics of the lymphoblastic cell lines used.

Subject ID	Sex	Age at Blood Collection	Age Onset	Clinical Presentation	c.709-1G > A Mutation Carrier
C8	M	46	-	Healthy	No
C13	M	70	-	Healthy	No
C18	F	58	-	Healthy	No
C20	M	69	-	Healthy	No
C22	F	64	-	Healthy	No
P5	F	70	64	FTD-bv; CBS	Yes
P10	F	70	65	PNFA; CBS	Yes
P25	F	65	63	FTD-bv; CBS	Yes
P30	F	54	54	FTD-bv	Yes
P31	F	70	63	FTD-bv; CBS	Yes

M: Male; F: Female; FTD-bv: Frontotemporal dementia (behavioral variant); CBS: Corticobasal Syndrome; PNFA: Progressive non fluent aphasia.

## Data Availability

Data is contained within the article and supplementary materials.

## Supplementary Materials

The following supporting information can be downloaded at: https://www.mdpi.com/article/10.3390/antiox12030581/s1. All data generated during this study are included in this published article and its supplementary information files. The raw datasets and western blot images are available from the corresponding authors upon reasonable request.

## Abbreviations

AD	Alzheimer’s disease.
ALS	Amyotrophic lateral sclerosis.
ANOVA	Analysis of variance
BafA1	Bafilomycin A1
BSA	Bovine serum albumin
BCA	Bicinchoninic acid
FCCP	Carbonylcyanide-p-trifluoromethoxyphenylhydrazone
CK-1δ	Casein kinase 1 delta
CxVβ	Complex V-β subunit
DAPI	4,6-diamidino-2phenylindole
DMEM	Dulbecco’s Modified Eagle Medium
Drp1	Dynamin-related protein 1
EBV	Epstein Barr virus
FBS	Fetal bovine serum
FIS1	Fission protein 1
FTLD-TDP	Frontotemporal lobar degeneration with TDP-43 inclusions
*GRN*	*GRN* gene
KD	Knockdown
LOF	Loss-of-function
Opa1	Mitochondrial dynamin-like GTPase
Mfn1	Mitofusin1
Mfn2	Mitofusin2
OCR	Oxygen consumption rate
PD	Parkinson’s disease
P/S.	Penicillin/streptomycin
PBS	Phosphate buffered saline
PGRN	Progranulin
PGC1α	Proliferator-activated receptor γ co-activator 1 α
ROS	Reactive Oxygen Species
SEM	Standard Error of the Mean
TDP-43	TAR DNA-binding protein 43
TMRE	Tetramethyl rhodamine ethyl ester
TMRM	Tetramethyl rhodamine methyl ester

## References

[1] Baker M., Mackenzie I.R., Pickering-Brown S.M., Gass J., Rademakers R., Lindholm C., Snowden J., Adamson J., Sadovnick A.D., Rollinson S. (2006). Mutations in Progranulin Cause Tau-Negative Frontotemporal Dementia Linked to Chromosome 17. Nature.

[2] Cruts M., Gijselinck I., van der Zee J., Engelborghs S., Wils H., Pirici D., Rademakers R., Vandenberghe R., Dermaut B., Martin J.-J. (2006). Null Mutations in Progranulin Cause Ubiquitin-Positive Frontotemporal Dementia Linked to Chromosome 17q21. Nature.

[3] Neumann M., Lee E.B., Mackenzie I.R. (2021). FTLD-TDP Pathological Subtypes: Clinical and Mechanistic Significance. Adv. Exp. Med. Biol..

[4] Natarajan K., Eisfeldt J., Hammond M., Laffita-Mesa J.M., Patra K., Khoshnood B., Öijerstedt L., Graff C. (2021). Single-Cell Multimodal Analysis in a Case with Reduced Penetrance of Progranulin-Frontotemporal Dementia. Acta Neuropathol. Commun..

[5] Dominguez J., Ng A., Yu J., Guevarra A.C., Daroy M.L., Alfon A., Catindig J.-A., Dizon M., Santiago J., Moral M.C.D. (2020). Autosomal Dominant Frontotemporal Lobar Degeneration in a Filipino Family with Progranulin Mutation. DEM.

[6] Hosaka T., Ishii K., Miura T., Mezaki N., Kasuga K., Ikeuchi T., Tamaoka A. (2017). A Novel Frameshift GRN Mutation Results in Frontotemporal Lobar Degeneration with a Distinct Clinical Phenotype in Two Siblings: Case Report and Literature Review. BMC Neurol..

[7] Cabezas-Opazo F.A., Vergara-Pulgar K., Pérez M.J., Jara C., Osorio-Fuentealba C., Quintanilla R.A. (2015). Mitochondrial Dysfunction Contributes to the Pathogenesis of Alzheimer’s Disease. Oxid. Med. Cell Longev..

[8] Johri A., Beal M.F. (2012). Mitochondrial Dysfunction in Neurodegenerative Diseases. J. Pharmacol. Exp. Ther..

[9] Stichel C.C., Zhu X.-R., Bader V., Linnartz B., Schmidt S., Lübbert H. (2007). Mono- and Double-Mutant Mouse Models of Parkinson’s Disease Display Severe Mitochondrial Damage. Hum. Mol. Genet..

[10] Wang W., Li L., Lin W.-L., Dickson D.W., Petrucelli L., Zhang T., Wang X. (2013). The ALS Disease-Associated Mutant TDP-43 Impairs Mitochondrial Dynamics and Function in Motor Neurons. Hum. Mol. Genet..

[11] Zhao J., Wang X., Huo Z., Chen Y., Liu J., Zhao Z., Meng F., Su Q., Bao W., Zhang L. (2022). The Impact of Mitochondrial Dysfunction in Amyotrophic Lateral Sclerosis. Cells.

[12] Dedert C., Mishra V., Aggarwal G., Nguyen A.D., Xu F. (2022). Progranulin Preserves Autophagy Flux and Mitochondrial Function in Rat Cortical Neurons Under High Glucose Stress. Front. Cell. Neurosci..

[13] Zhou D., Zhou M., Wang Z., Fu Y., Jia M., Wang X., Liu M., Zhang Y., Sun Y., Lu Y. (2019). PGRN Acts as a Novel Regulator of Mitochondrial Homeostasis by Facilitating Mitophagy and Mitochondrial Biogenesis to Prevent Podocyte Injury in Diabetic Nephropathy. Cell Death Dis..

[14] de la Encarnación A., Alquézar C., Esteras N., Martín-Requero Á. (2015). Progranulin Deficiency Reduces CDK4/6/PRb Activation and Survival of Human Neuroblastoma SH-SY5Y Cells. Mol. Neurobiol..

[15] de la Encarnación A., Alquézar C., Martín-Requero Á. (2016). Increased Wnt Signaling and Reduced Viability in a Neuronal Model of Progranulin-Deficient Frontotemporal Lobar Degeneration. Mol. Neurobiol..

[16] Alquezar C., Esteras N., Alzualde A., Moreno F., Ayuso M.S., de Munain A.L., Martín-Requero Á. (2012). Inactivation of CDK/PRb Pathway Normalizes Survival Pattern of Lymphoblasts Expressing the FTLD-Progranulin Mutation c.709-1G>A. PLoS ONE.

[17] Gao X., Joselin A.P., Wang L., Kar A., Ray P., Bateman A., Goate A.M., Wu J.Y. (2010). Progranulin Promotes Neurite Outgrowth and Neuronal Differentiation by Regulating GSK-3β. Protein Cell.

[18] Alquezar C., Salado I.G., De La Encarnación A., Pérez D.I., Moreno F., Gil C., De Munain A.L., Martínez A., Martín-Requero Á. (2016). Targeting TDP-43 Phosphorylation by Casein Kinase-1δ Inhibitors: A Novel Strategy for the Treatment of Frontotemporal Dementia. Mol. Neurodegener..

[19] Salado I.G., Redondo M., Bello M.L., Perez C., Liachko N.F., Kraemer B.C., Miguel L., Lecourtois M., Gil C., Martinez A. (2014). Protein Kinase CK-1 Inhibitors As New Potential Drugs for Amyotrophic Lateral Sclerosis. J. Med. Chem..

[20] Alquezar C., Esteras N., Bartolomé F., Merino J.J., Alzualde A., de Munain A.L., Martín-Requero Á. (2012). Alteration in Cell Cycle-Related Proteins in Lymphoblasts from Carriers of the c.709-1G>A PGRN Mutation Associated with FTLD-TDP Dementia. Neurobiol. Aging.

[21] Alquezar C., Esteras N., de la Encarnación A., Moreno F., de Munain A.L., Martín-Requero Á. (2015). Increasing Progranulin Levels and Blockade of the ERK1/2 Pathway: Upstream and Downstream Strategies for the Treatment of Progranulin Deficient Frontotemporal Dementia. Eur. Neuropsychopharmacol..

[22] Bartolome F., Wu H.-C., Burchell V.S., Preza E., Wray S., Mahoney C.J., Fox N.C., Calvo A., Canosa A., Moglia C. (2013). Pathogenic VCP Mutations Induce Mitochondrial Uncoupling and Reduced ATP Levels. Neuron.

[23] Oleinik N., Kim J., Roth B.M., Selvam S.P., Gooz M., Johnson R.H., Lemasters J.J., Ogretmen B. (2019). Mitochondrial Protein Import is Regulated by P17/PERMIT to Mediate Lipid Metabolism and Cellular Stress. Sci. Adv..

[24] Imamura H., Huynh Nhat K.P., Togawa H., Saito K., Iino R., Kato-Yamada Y., Nagai T., Noji H. (2009). Visualization of ATP Levels Inside Single Living Cells with Fluorescence Resonance Energy Transfer-Based Genetically Encoded indicators. Proc. Natl. Acad. Sci. USA.

[25] de la Cueva M., Antequera D., Ordoñez-Gutierrez L., Wandosell F., Camins A., Carro E., Bartolome F. (2022). Amyloid-β Impairs Mitochondrial Dynamics and Autophagy in Alzheimer’s Disease Experimental Models. Sci. Rep..

[26] Bartolome F., de la Cueva M., Pascual C., Antequera D., Fernandez T., Gil C., Martinez A., Carro E. (2018). Amyloid β-Induced Impairments on Mitochondrial Dynamics, Hippocampal Neurogenesis, and Memory are Restored by Phosphodiesterase 7 Inhibition. Alzheimers Res. Ther..

[27] Bartolome F., Antequera D., Tavares E., Pascual C., Maldonado R., Camins A., Carro E. (2017). Obesity and Neuroinflammatory Phenotype in Mice Lacking Endothelial Megalin. J. Neuroinflamm..

[28] Guo A., Tapia L., Bamji S.X., Cynader M.S., Jia W. (2010). Progranulin Deficiency Leads to Enhanced Cell Vulnerability and TDP-43 Translocation in Primary Neuronal Cultures. Brain Res..

[29] Valdez C., Wong Y.C., Schwake M., Bu G., Wszolek Z.K., Krainc D. (2017). Progranulin-Mediated Deficiency of Cathepsin D Results in FTD and NCL-Like Phenotypes in Neurons Derived from FTD Patients. Hum. Mol. Genet..

[30] Li Q., Yokoshi M., Okada H., Kawahara Y. (2015). The Cleavage Pattern of TDP-43 Determines Its Rate of Clearance and Cytotoxicity. Nat. Commun..

[31] Phan K., He Y., Pickford R., Bhatia S., Katzeff J.S., Hodges J.R., Piguet O., Halliday G.M., Kim W.S. (2020). Uncovering Pathophysiological Changes in Frontotemporal Dementia Using Serum Lipids. Sci. Rep..

[32] Yousef A., Robinson J.L., Irwin D.J., Byrne M.D., Kwong L.K., Lee E.B., Xu Y., Xie S.X., Rennert L., Suh E. (2017). Neuron Loss and Degeneration in the Progression of TDP-43 in Frontotemporal Lobar Degeneration. Acta. Neuropathol. Commun..

[33] López de Munain A., Alzualde A., Gorostidi A., Otaegui D., Ruiz-Martínez J., Indakoetxea B., Ferrer I., Pérez-Tur J., Sáenz A., Bergareche A. (2008). Mutations in Progranulin Gene: Clinical, Pathological, and Ribonucleic Acid Expression Findings. Biol. Psychiatry.

[34] Geisler S., Holmström K.M., Skujat D., Fiesel F.C., Rothfuss O.C., Kahle P.J., Springer W. (2010). PINK1/Parkin-Mediated Mitophagy is Dependent on VDAC1 and P62/SQSTM1. Nat. Cell Biol..

[35] Tanaka Y., Suzuki G., Matsuwaki T., Hosokawa M., Serrano G., Beach T.G., Yamanouchi K., Hasegawa M., Nishihara M. (2017). Progranulin Regulates Lysosomal Function and Biogenesis through Acidification of Lysosomes. Hum. Mol. Genet..

[36] Chang M.C., Srinivasan K., Friedman B.A., Suto E., Modrusan Z., Lee W.P., Kaminker J.S., Hansen D.V., Sheng M. (2017). Progranulin Deficiency Causes Impairment of Autophagy and TDP-43 Accumulation. J. Exp. Med..

[37] Yoshii S.R., Mizushima N. (2017). Monitoring and Measuring Autophagy. Int. J. Mol. Sci..

[38] Kametani F., Nonaka T., Suzuki T., Arai T., Dohmae N., Akiyama H., Hasegawa M. (2009). Identification of Casein Kinase-1 Phosphorylation Sites on TDP-43. Biochem. Biophys. Res. Commun..

[39] Gao J., Wang L., Yan T., Perry G., Wang X. (2019). TDP-43 Proteinopathy and Mitochondrial Abnormalities in Neurodegeneration. Mol. Cell. Neurosci..

[40] Wang W., Wang L., Lu J., Siedlak S.L., Fujioka H., Liang J., Jiang S., Ma X., Jiang Z., da Rocha E.L. (2016). The Inhibition of TDP-43 Mitochondrial Localization Blocks Its Neuronal Toxicity. Nat. Med..

[41] Wang P., Deng J., Dong J., Liu J., Bigio E.H., Mesulam M., Wang T., Sun L., Wang L., Lee A.Y.-L. (2019). TDP-43 Induces Mitochondrial Damage and Activates the Mitochondrial Unfolded Protein Response. PLoS Genet..

[42] Rabinovici G.D., Miller B.L. (2010). Frontotemporal Lobar Degeneration: Epidemiology, Pathophysiology, Diagnosis and Management. CNS Drugs.

[43] Bartolome F., Esteras N., Martin-Requero A., Boutoleau-Bretonniere C., Vercelletto M., Gabelle A., Le Ber I., Honda T., Dinkova-Kostova A.T., Hardy J. (2017). Pathogenic P62/SQSTM1 Mutations Impair Energy Metabolism through Limitation of Mitochondrial Substrates. Sci. Rep..

[44] Bobba A., Amadoro G., Valenti D., Corsetti V., Lassandro R., Atlante A. (2013). Mitochondrial Respiratory Chain Complexes I and IV are Impaired by β-Amyloid via Direct Interaction and through Complex I-Dependent ROS Production, respectively. Mitochondrion.

[45] Gandhi S., Wood-Kaczmar A., Yao Z., Plun-Favreau H., Deas E., Klupsch K., Downward J., Latchman D.S., Tabrizi S.J., Wood N.W. (2009). PINK1-Associated Parkinson’s Disease Is Caused by Neuronal Vulnerability to Calcium-Induced Cell Death. Mol. Cell.

[46] Su J.H., Nichol K.E., Sitch T., Sheu P., Chubb C., Miller B.L., Tomaselli K.J., Kim R.C., Cotman C.W. (2000). DNA Damage and Activated Caspase-3 Expression in Neurons and Astrocytes: Evidence for Apoptosis in Frontotemporal Dementia. Exp. Neurol..

[47] Morán M., Rivera H., Sánchez-Aragó M., Blázquez A., Merinero B., Ugalde C., Arenas J., Cuezva J.M., Martín M.A. (2010). Mitochondrial Bioenergetics and Dynamics Interplay in Complex I-Deficient Fibroblasts. Biochim. Biophys. Acta BBA Mol. Basis Dis..

[48] Abramov A.Y., Angelova P.R. (2019). Cellular Mechanisms of Complex I-Associated Pathology. Biochem. Soc. Trans..

[49] Ma K., Chen G., Li W., Kepp O., Zhu Y., Chen Q. (2020). Mitophagy, Mitochondrial Homeostasis, and Cell Fate. Front. Cell Dev. Biol..

[50] Elmore S.P., Qian T., Grissom S.F., Lemasters J.J. (2001). The Mitochondrial Permeability Transition Initiates Autophagy in Rat Hepatocytes. FASEB J..

[51] Priault M., Salin B., Schaeffer J., Vallette F.M., di Rago J.-P., Martinou J.-C. (2005). Impairing the Bioenergetic Status and the Biogenesis of Mitochondria Triggers Mitophagy in Yeast. Cell Death Differ..

[52] Twig G., Elorza A., Molina A.J.A., Mohamed H., Wikstrom J.D., Walzer G., Stiles L., Haigh S.E., Katz S., Las G. (2008). Fission and Selective Fusion Govern Mitochondrial Segregation and Elimination by Autophagy. EMBO J..

[53] Xu Y.-F., Gendron T.F., Zhang Y.-J., Lin W.-L., D’Alton S., Sheng H., Casey M.C., Tong J., Knight J., Yu X. (2010). Wild-Type Human TDP-43 Expression Causes TDP-43 Phosphorylation, Mitochondrial Aggregation, Motor Deficits, and Early Mortality in Transgenic Mice. J. Neurosci..

[54] Alquézar C., Esteras N., de la Encarnación A., Alzualde A., Moreno F., López de Munain A., Martín-Requero Á. (2014). PGRN Haploinsufficiency Increased Wnt5a Signaling in Peripheral Cells from Frontotemporal Lobar Degeneration-Progranulin Mutation Carriers. Neurobiol. Aging.

[55] Alquézar C., de la Encarnación A., Moreno F., López de Munain A., Martín-Requero Á. (2016). Progranulin Deficiency Induces Overactivation of WNT5A Expression via TNF-α/NF-ΚB Pathway in Peripheral Cells from Frontotemporal Dementia-Linked Granulin Mutation Carriers. J. Psychiatry Neurosci..

[56] Hu Q., Ansari M., Angelidis I., Theis F., Schiller H., Königshoff M., Lehmann M. (2021). Chronic WNT/ß-Catenin Signaling Impairs Mitochondrial Function in Lung Epithelial Cells. Eur. Respir. J..

[57] Silva-Alvarez C., Arrazola M., Godoy J., Ordenes D., Inestrosa N. (2013). Canonical Wnt Signaling Protects Hippocampal Neurons from Aβ Oligomers: Role of Non-Canonical Wnt-5a/Ca^2+^ in Mitochondrial Dynamics. Front. Cell. Neurosci..

[58] Yoon J.C., Ng A., Kim B.H., Bianco A., Xavier R.J., Elledge S.J. (2010). Wnt Signaling Regulates Mitochondrial Physiology and Insulin Sensitivity. Genes. Dev..

[59] Costa R., Peruzzo R., Bachmann M., Montà G.D., Vicario M., Santinon G., Mattarei A., Moro E., Quintana-Cabrera R., Scorrano L. (2019). Impaired Mitochondrial ATP Production Downregulates Wnt Signaling via ER Stress Induction. Cell Rep..

[60] Delgado-Deida Y., Alula K.M., Theiss A.L. (2020). The Influence of Mitochondrial-Directed Regulation of Wnt Signaling on Tumorigenesis. Gastroenterol. Rep..

