# Supplementary material for: Progranulin Deficiency Induces Mitochondrial Dysfunction in Frontotemporal Lobar Degeneration with TDP-43 Inclusions
Source: Antioxidants (Basel). 2023 Feb 25;12(3):581. doi: 10.3390/antiox12030581 (PMC10044829; doi:10.3390/antiox12030581)
Supplement: Supplementary file 1 [file antioxidants-12-00581-s001.zip › antioxidants-2188036-supplementary.pdf]

Supplementary Material

Progranulin deficiency induces mitochondrial dysfunction in FTLD-TPD

Guiomar Rodríguez-Periñán, Ana de la Encarnación, Fermín Moreno, Adolfo López de Munain, Ana Martínez, Ángeles Martín-Requero, Carolina Alquézar[†], Fernando Bartolomé[†].

[†]Corresponding authors:

Dr Fernando Bartolome
Group of Neurodegenerative Diseases
Hospital 12 de Octubre Research Institute
28041-Madrid, Spain
Phone: +34 913908765
Fax: +34 913908544
E-mail: fbartolome.imas12@h12o.es
ORCID: 0000-0001-9322-8933

Dr Carolina Alquezar
Group of Neurodegenerative Diseases
Hospital 12 de Octubre Research Institute
28041-Madrid, Spain
Phone: +34 913908765
Fax: +34 913908544
E-mail: carolinaalquezar.imas12@h12o.es
ORCID: 0000-0002-7602-310

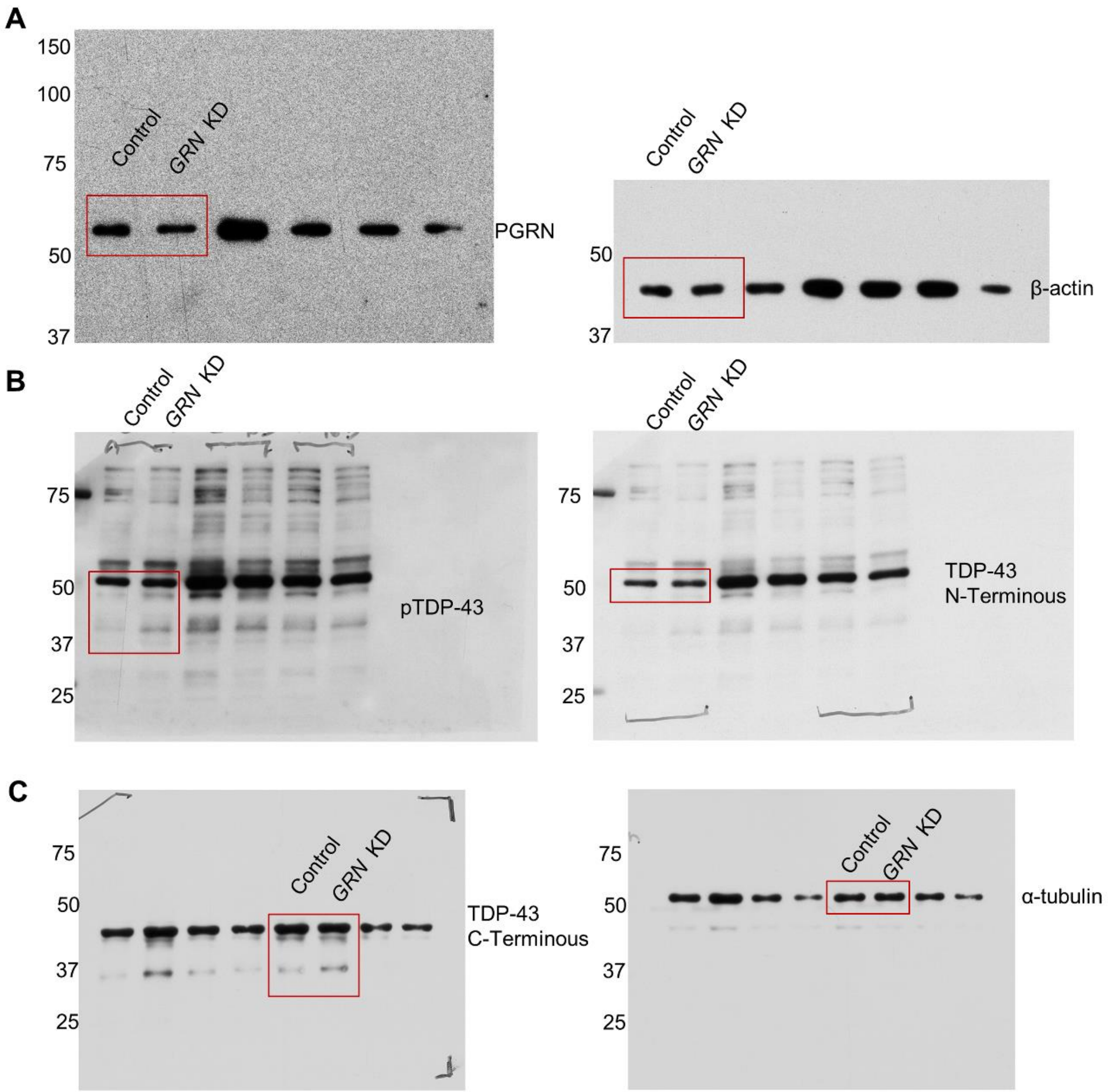

Supplementary Figure S1. Full-length blots from Figure 1. A) PGRN and β-actin immunoblots. The membrane was cut below 75kD after the incubation with the anti-PGRN antibody and before the incubation the anti-actin antibody. B) pTDP-43 and total TDP-43 immunoblots. The membrane was cut at 75 kD and the bottom part was incubated with an anti Phospho-TDP43 (Ser409/410) Polyclonal antibody. Then, membrane was stripped and incubated with an anti N-Terminal TDP43 antibody that recognizes the full-length protein. C) TDP-43 C-Terminous (CT) and total α-tubulin immunoblots. The membrane was cut at 75 kD. The bottom part of the membrane was incubated first with an anti TDP43 antibody recognizing the CT region of the protein and then stripped and incubated with an anti-tubulin antibody that was used as loading control. Red Boxes frame the representative bands showed in the main figure 1.

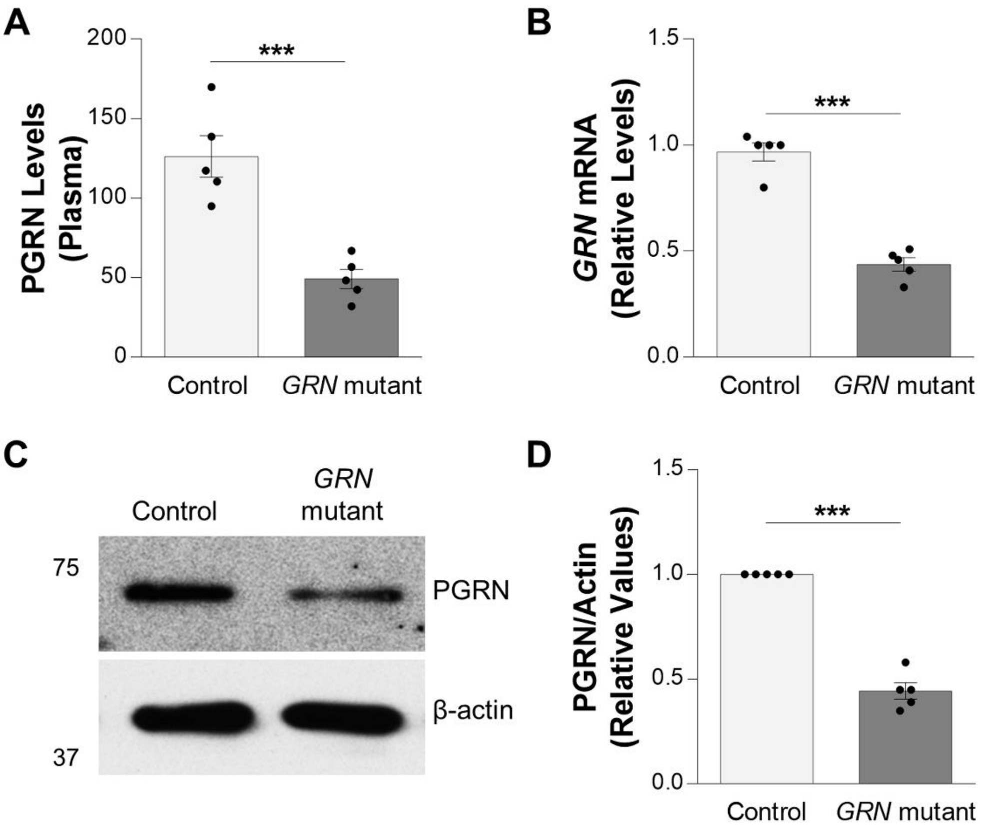

Supplementary Figure S2. Heterozygous c.709-1G>A *GRN* mutation induces PGRN happloinsufficiency in FTLD-TDP patients. A) Plasma PGRN levels in FTLD-TDP patients carrying the c.709-1G>A *GRN* mutation and age and sex matched healthy controls. B) Immortalized lymphoblasts from FTLD-TDP patients carrying the c.709-1G>A *GRN* mutation show decreased *GRN* mRNA, compared with lymphoblasts derived from healthy controls C) Representative image showing PGRN levels in lymphoblasts from control subjects and FTLD-TDP patients carrying the c.709-1G>A *GRN* mutation. β-actin was used as loading control. D) Plot representing the average ± SEM of PGRN levels measured in all the subjects used in this study. The statistical analysis was performed using Student's t-test. ***p < 0.001.

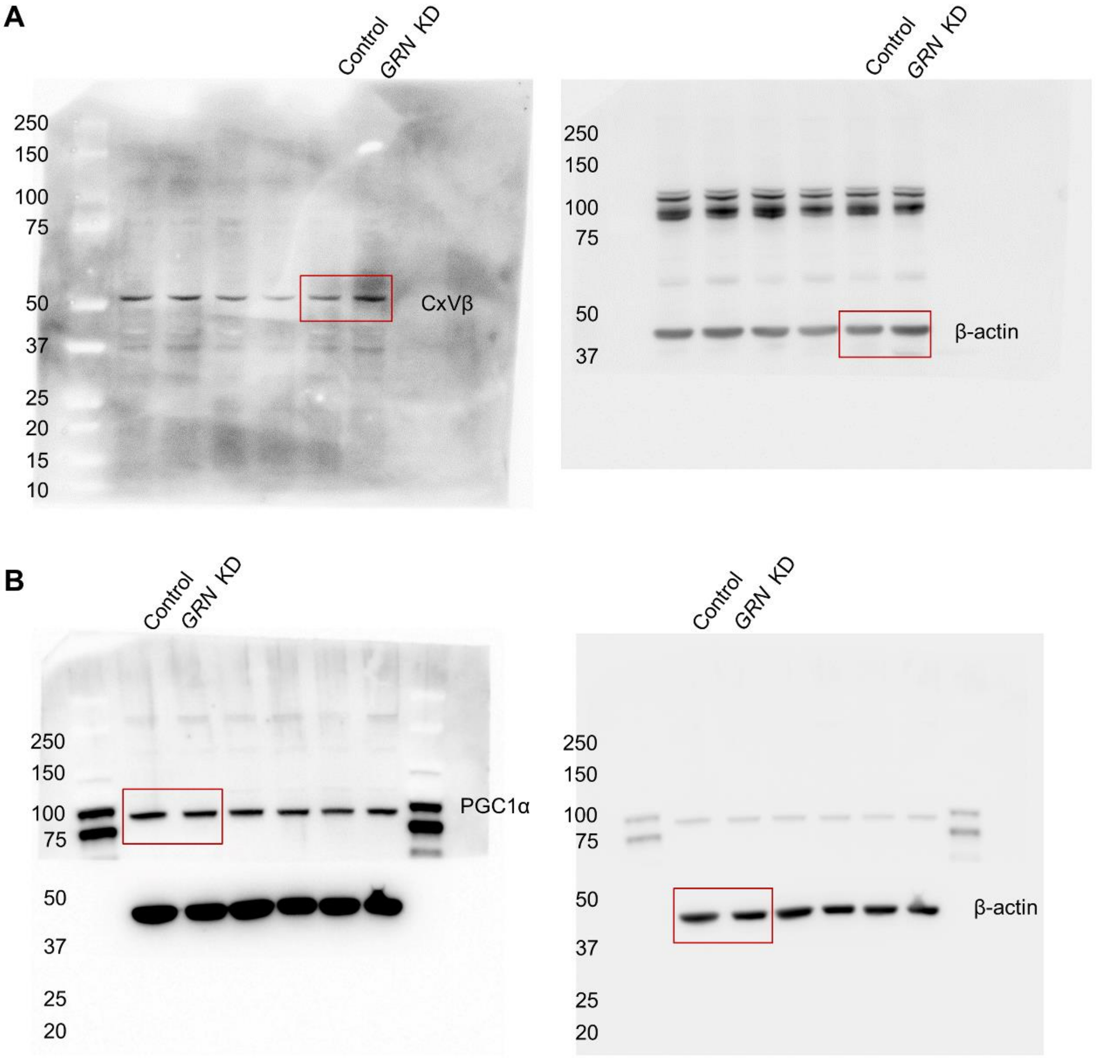

Supplementary Figure S3. Full-length blots from Figure 3. A) CxVβ and β-actin immunoblot. After the incubation with an anti-CxVβ the m embrane was cut below 37kD and then incubated with an antibody recognizing β-actin. The estimated molecular weight (MW) of CxVβ and β-actin are 52kD and 43kD, respectively. The bands running at other MW are remains of previous incubations. B) PGC1α and β-actin immunoblots. The membrane was cut below 75 kD. Top part of the membrane was incubated with an anti-PGC1α antibody (MW≈ 91kD) and bottom part was incubated with an antibody recognizing β-actin. Left panel shows the high exposure image and right part shows a low exposure image. Red Boxes frame the representative bands showed in the main figure 3.

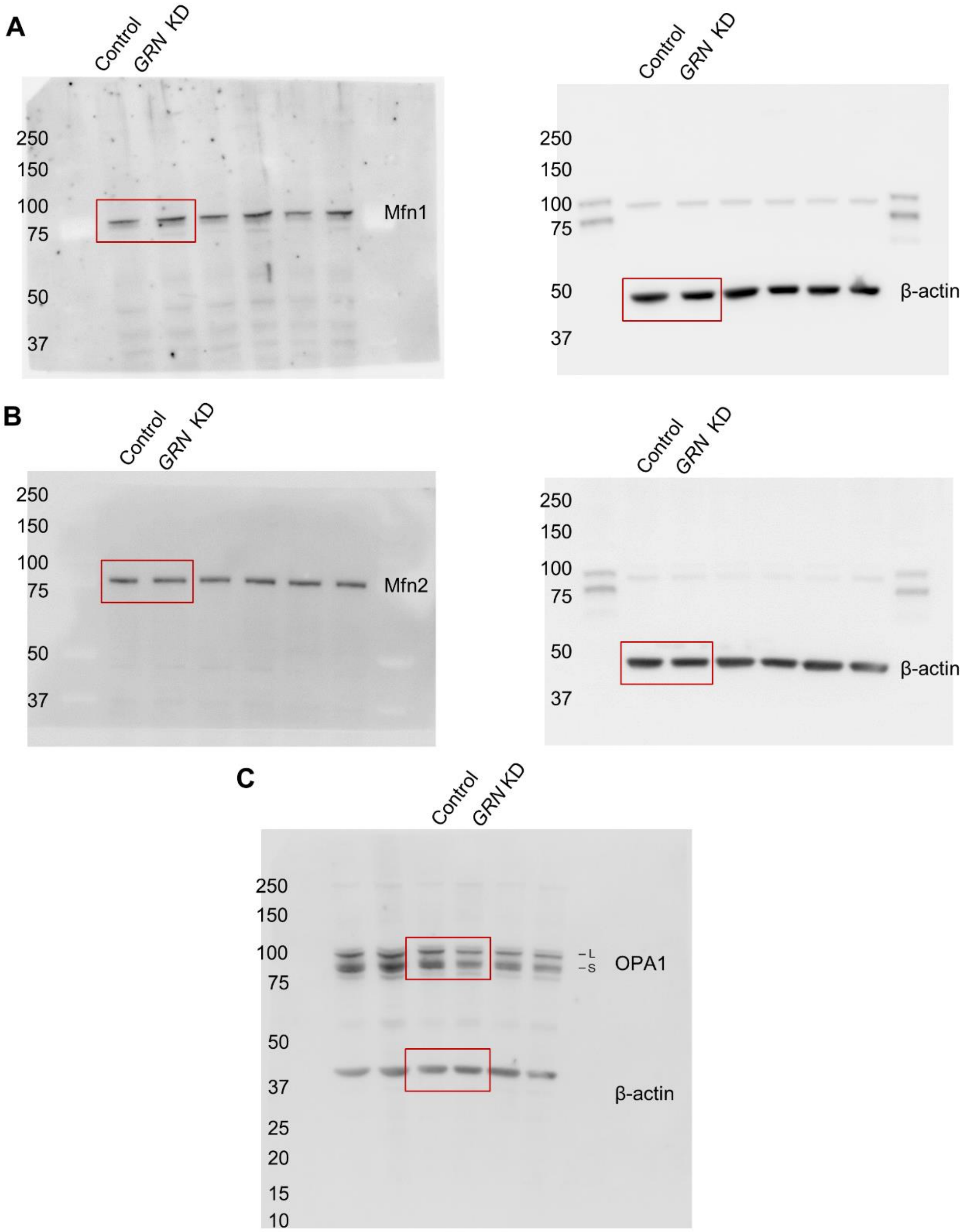

Supplementary Figure S4. Full-length blots from Figure 4A. A) Mfn1and β-actin immunoblot. This membrane was cut below 37kD before the incubation with anti-Mfn1 and anti-β-actin antibodies. B) Mfn2 and β-actin immunoblots. The membrane was cut at 37kD before the incubation with both antibodies. C) OPA1 and β-actin immunoblot. Full membrane was incubated with anti-OPA1 and anti-β-actin antibodies. The estimated molecular weight (MW) of the proteins assessed in this figure are 80kD for Mfn1/2, 43kD for β-actin and 98kD and 85kD for OPA1 L and OPA1 S, respectively. The bands running at other MW are remains of previous incubations. Red Boxes frame the representative bands showed in the main figure 4A.

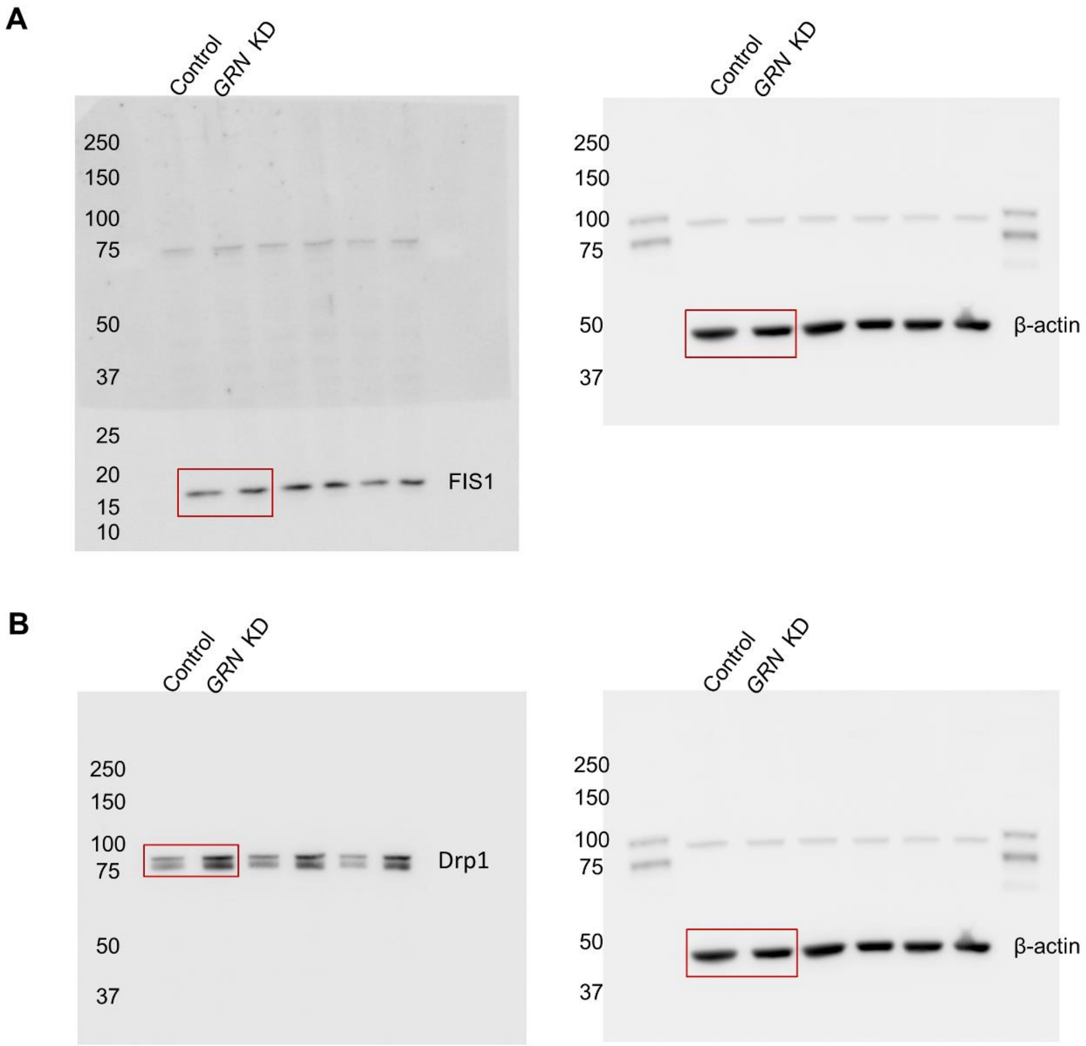

Supplementary Figure S5. Full-length blots from Figure 4B. A) FIS1 and β-actin immunoblots. This membrane was cut at 37kD. The bottom part was incubated with anti-FIS1 antibody (MW≈17kD) and the top part was incubated with several other antibodies before the incubation with an anti-β-actin antibody. The estimated MW of β-actin is 43kD. The faint bands running at other MW are remains of previous incubations. B) Drp1 and β-actin immunoblots. The membrane was cut below 37kD and the top part of the membrane was incubated first with an anti-Drp1 antibody (left panel) and then striped and incubated with an anti- β-actin antibody (right panel). The faint top band of the right panel is a remaining of a previous incubation using an anti-PGC1α antibody. Red Boxes frame the representative bands showed in the main figure 4B.

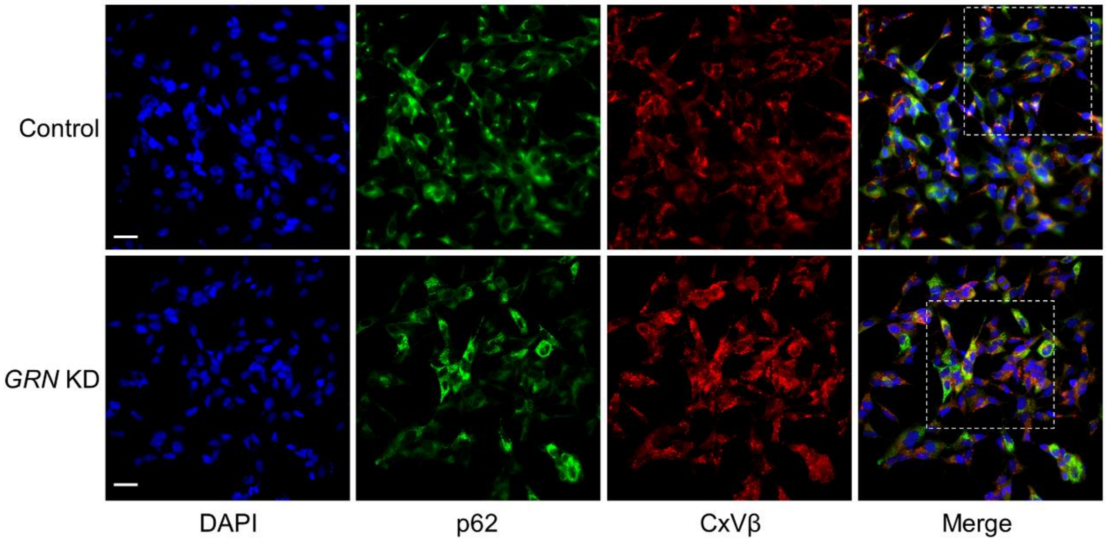

Supplementary Figure S6. Representative images showing DAPI (blue), p62 (green) and CxVβ (red) staining in control and *GRN* KD SH-SY5Y cells. Images were acquired using a confocal microscope with a 40× water-immersion objective. Scale bar = 9µm. The dashed square represents the region of interest used in main Figure 4.

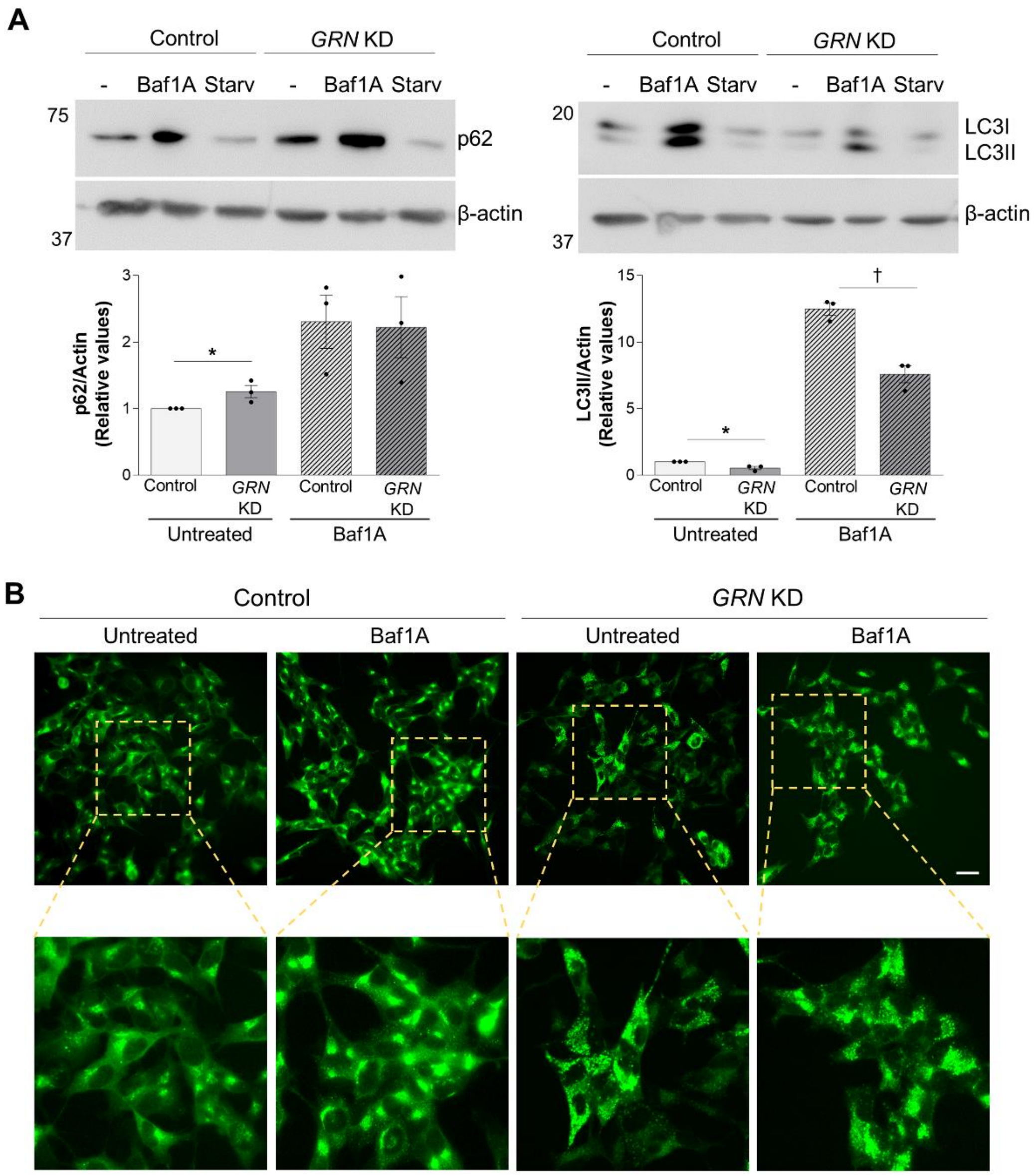

Supplementary Figure S7. PGRN deficiency induces autophagy impairment in SH-SY5Y cells. A) Representative immunoblots showing that PGRN deficient SH-SY5Y cells display higher p62 levels and lower LC3II levels, compared with control cells. 100 nM Baf1A treatment increased LC3II levels in both control and *GRN* KD cells. However, the increase of LC3II levels was not as evident in PGRN deficient cells implying an autophagy impairment associated with PGRN deficiency. β-actin was used as loading control. Plots represent the average ± SEM of three independent measurements of p62 and LC3II levels, respectively. Statistical analysis was performed using two-way ANOVA´s tests followed by Bonferroni´s correction. *$p < 0.05$ significant difference between untreated control and *GRN* KD cells. †$p < 0.01$ significant difference between Baf1A treated cells. B) p62 immunofluorescence (green) in control and *GRN* KD SH-SY5Y cells before and after Baf1A treatment. In basal conditions, p62 staining showed a diffuse distribution in control cells and puncta distribution in *GRN* KD cells. Baf1A treatment blocked autophagy in control cells leading to the accumulation of p62 puncta near the nuclei. Baf1A treatment did not affect p62 distribution in PGRN deficient cells, confirming that autophagy was impaired in these cells. Images were acquired using a confocal microscope with a 40× water-immersion objective. Scale bar = 9µm.
